# Development of Pd-Loaded Hf-Based Metal–Organic Framework as a Dual-Modal Contrast Agent for Photoacoustic Imaging and Computed Tomography

**DOI:** 10.1021/acsbiomaterials.5c00169

**Published:** 2025-05-06

**Authors:** Yen-Chang Chen, Yu-Sheng Yu, Yu-Kang Wang, R. K. Rakesh Kumar, Cho-yin Lee, Cheng-Hsin Chuang, Lun-De Liao, Kevin C.-W. Wu

**Affiliations:** † Department of Chemical Engineering, National Taiwan University, Taipei 10617, Taiwan; ‡ Institute of Biomedical Engineering and Nanomedicine, National Health Research Institutes, Miaoli 350401, Taiwan; § International Graduate Program of Molecular Science and Technology, Taiwan International Graduate Program, Academia Sinica, Taipei 115201, Taiwan; ∥ International Graduate Program of Molecular Science and Technology (NTU-MST), National Taiwan University, Taipei 10617, Taiwan; ⊥ Department of Biomedical Engineering, National Yang Ming Chiao Tung University, Taipei 30010, Taiwan; # Department of Radiation Oncology, Taoyuan General Hospital, Ministry of Health and Welfare, Taoyuan 330215, Taiwan; ¶ Institute of Medical Science and Technology, National Sun Yat-sen University, Kaohsiung 804201, Taiwan; & Department of Chemical Engineering and Materials Science, Yuan Ze University, Chung-Li, Taoyuan 32003, Taiwan

**Keywords:** contrast agent, CT/PA dual-modal imaging, metal−organic framework, palladium nanoparticles

## Abstract

Noninvasive cancer imaging significantly improves diagnostics by providing comprehensive structural and functional information about tumors. Herein, we explored palladium nanoparticles loaded hafnium-based metal–organic framework (MOF) (Hf-EDB), i.e., Pd@Hf-EDB as an efficient dual modal contrast agent for computed tomography (CT) and photoacoustic imaging (PAI). The synergistic collaborations between (i) high-Z element Hf-based MOF with superior X-rays absorbing capabilities, (ii) H_2_EDB linkers with special π-donation and π-acceptor characteristics capable of strongly anchoring noble metals, and (iii) Pd nanoparticles with broad absorption in the UV to near-infrared (NIR) regions due to strong interband transition are ideal for implementation in CT and PAI. The successful synthesis of Pd@Hf-EDB nanoparticles was confirmed through morphology, crystallinity, and compositional characterizations using X-ray diffraction, SEM, TEM, DLS, and EDS. Soft X-ray tomography verified cellular uptake via phagocytosis of Pd@Hf-EDB by BxPC-3 tumor cells. In-vitro experiments revealed superior CT imaging performance of Pd@Hf-EDB over traditional molecular contrast agents like Iohexol. Broad absorption range in the UV–vis/NIR regions and superior PAI capabilities of Pd@Hf-EDB relative to gold nanorods are reported. Furthermore, the in vivo xenograft model demonstrated significant contrast enhancements near the tumor, highlighting the excellent PAI and CT capabilities of the synthesized Pd@Hf-EDB.

## Graphical abstract

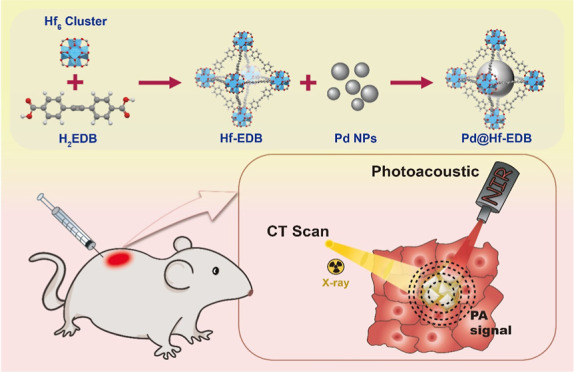

## 1 Introduction

Noninvasive cancer imaging aimed at providing structural and functional information about tumors has become essential in clinical care as it can significantly improve diagnostic accuracy.^1–4^ Noninvasive imaging substantially lowers the risk of complications by reducing unnecessary surgical procedures, enhancing patient comfort, and developing personalized treatment(s).^4–6^ Recently, noninvasive imaging techniques, including positron emission tomography (PET),^7,8^ magnetic resonance imaging (MRI),^9^ computed tomography (CT),^10^ single-photon emission computed tomography (SPECT),^11^ and ultrasound^12,13^ are being extensively utilized in clinical practice. Driven by an ever-increasing need for improvements in reliability and accuracy, newer technologies have emerged including photoacoustic imaging (PAI).^14^ As such, different imaging modalities possessing unique combinations of capabilities and functions are developed and tailored to suit specific conditions. Many contrast agents with multimodal imaging capabilities are being investigated to harness the advantages of various imaging techniques and overcome the limitations of single-modality imaging. For instance, Cai et al.^15^ reported on a dual-function PET and near-infrared (NIR) fluorescence probe for tumor vasculature imaging. Zhang et al.^16^ developed Gd/CuS-loaded nanogels to enable MR/PA dual-mode imaging-guided photothermal therapy. Song et al.^17^ investigated multimodal image fusion methods using MRI and PET to improve Alzheimer’s disease diagnosis. Conclusively, multimodal imaging approach offers several benefits, such as obtaining complementary diagnostic information at the reduced expense of reduced dosage and frequency of contrast agent use.^15,18,19^

CT is a widely used diagnostic tool that provides detailed cross-sectional images of the body and high-resolution anatomical information with rapid imaging capabilities.^20^ CT relies on the differential absorption of X-rays by various tissues, often using contrast agents to enhance image quality.^20,21^ On the other hand, PAI is a relatively new imaging modality that combines the high contrast of optical imaging with the high spatial resolution of ultrasound.^22–25^ PAI utilizes the PA effect generated when an optical absorber such as hemoglobin is induced by a laser pulse, causing thermoelastic expansion thereby generating ultrasound waves (PA signals) that can be detected to form optical images.^26,27^ A significant advantage of PAI in tumor imaging is its ability to provide functional and molecular information on the tissues,^28^ such as tumor angiogenesis^29^ and hypoxia in various types of tumors.^13,30^ Furthermore, imaging in the NIR range, allows deeper tissue penetration and reduces scattering, making it ideal for in-depth imaging of body structures.^24,31,32^ However, the PA capability is inherently limited by the blood supply around the tumor, particularly for small or poorly vascularized early tumors. Thus, the use of contrast agents can provide more functional and molecular information, improving the accuracy and complexity of tumor monitoring.^28,33^ To this end, nanoparticles are well suited for designing multimodal contrast agents due to their excellent potential for functionalization.^34,35^ They can effectively combine diverse and specialized properties from different materials to produce desired aptitudes, including enhanced imaging capabilities and amendabilty towards therapeutic applications.

Consequently, nanomaterial augmented dual-modal contrast agents capable of harnessing the advantages of both CT and PAI techniques have been reported in recent years. For instance, Orza et al. developed an Au-Agl nanocomposite as a dual-modal contrast agent which could simultaneously enhance both CT and PA imaging.^36^ Additionally, highly porous materials such as metal–organic frameworks (MOFs) composed of metal clusters and organic linkers are exceptionally apt as proficient nanocarriers. MOFs owing to unique advantageous properties including high porosity, tunable pore size, and surface functionalities can be efficiently tailored to meet the ideal characteristics for imaging contrast agents and delivering drugs.^37–39^ They are constructed via coordination bonds between various metal ions or secondary building units and organic ligands, allowing for the formation of different MOFs.

In this work, we developed a novel dual-modal contrast agent for CT and PAI, by combining Pd nanoparticles (Pd NPs) and Hf-based MOF. Hf-based MOF (Hf-EDB) composed of Hf_6_O_4_(OH)_4_ clusters and 4,4′-(ethyne-1,2-diyl) dibenzoic acid (H_2_EDB) linker was strategically chosen as the nanocarrier. The Hf is a high-Z element, which can absorb more X-rays than soft tissue. Consequently, when precisely accumulated in the tumor site, it will have a higher contrast for X-ray than the healthy tissue, thus functioning as a good CT contrast agent. Furthermore, the ethynyl groups on the H_2_EDB linkers have special π-donation and π-acceptor characteristics that can strongly interact with the noble metal ions.^40^ Previously, we have reported that Pd^2+^ can adsorb onto the ethynyl groups from other linkers in the pores of Hf-PEB and reduce them into Pd NPs.^41^ Pd NPs can be effectively loaded into the pores of Hf-EDB in the same way to produce Pd@Hf-EDB. Moreover, these loaded Pd NPs exhibit strong interband transition absorption that provides a broad absorption from the UV to NIR regions,^42^ enabling them to be ideal contrast agent for PAI in NIR regions. Compared to traditional molecular contrast agents, metallic nanoparticles offer several advantages.^35,43,44^ These nanoparticles generally have longer circulation times, higher stability, and can achieve the same imaging effect at lower doses when used in vivo.^35,45^ Their ease of functionalization is extremely beneficial for targeted imaging, providing excellent sensitivity and specificity. In particular, traditional iodine-based contrast agents employed for CT have limitations including short circulation time and potential nephrotoxicity, which is a concern for patients with pre-existing kidney conditions.^46–48^ Therefore, we demonstrated that the synthesized Pd@Hf-EDB possessed dual-modal imaging capacities. It exhibited higher X-ray absorbance than iodine-based contrast agents and possessed superior imaging capabilities as compared to gold nanorods (GNRs) owing to Pd NPs with stability than GNRs after long-term laser irradiation.^18^ These results show that Pd@Hf-EDB possesses excellent imaging capabilities for both CT and PAI.

## 2 Experimental Section

### 2.1 Synthesis of 4,4′-(Ethyne-1,2-diyl)­dibenzoic Acid (H_2_EDB) Linker

The synthesis of H_2_EDB can be divided into two parts, (1) the synthesis of precursor bis­(4-(methoxycarbonyl)­phenyl)­acetylene (Me_2_EDB) through Sonogashira cross-coupling reaction (Figure S1a) and (2) the subsequent hydrolysis and acidification of Me_2_EDB to form 4,4′-(ethyne-1,2-diyl)­dibenzoic acid (H_2_EDB) (Figure S1b).^49,50^

#### 2.1.1 Synthesis of Me_2_EDB

Methyl 4-iodobenzoate (2.62 g, 10.0 mmol) and methyl 4-ethynylbenzoate (1.602 g, 10.0 mmol) were added to a three-neck round-bottomed flask and dissolved in 50 mL of TEA/toluene mixture (v/v = 1:1) under magnetic stirring. The mixture was degassed in vacuum and stirred for 10 min before adding bis-triphenylphosphine-palladium­(II) chloride (PdCl_2_(PPh_3_)_2_) (176 mg, 0.25 mmol) and copper­(I) iodide (10.0 mg, 0.05 mmol). Then, the mixture was stirred for 24 h at room temperature under nitrogen flow. Next, the reaction mixture was filtered and washed sequentially with copious amounts of hexane, saturated NH_4_Cl aqueous solution, saturated NaCl aqueous solution, and deionized (DI) water. The product was partially dried and redispersed in 30 mL of DCM. This mixture was stirred for at least 2 h, collected by filtration, and dried overnight in a vacuum oven.

#### 2.1.2 Synthesis of H_2_EDB

Me_2_EDB (0.639 g, 2.40 mmol) was suspended in 175 mL of a methanol/tetrahydrofuran mixture (v/v = 1:1). Then, 150 mL of DI water containing potassium hydroxide (1.346 g, 24.00 mmol) was added. The mixture was stirred and refluxed in a 75 °C oil bath. The resulting solution was allowed to cool to a temperature below 40 °C. And the product (i.e., H_2_EDB) was precipitated by adding 10 mL of HCl to the mixture. The product was then collected via centrifugation, following several rounds of sonication and washing to remove the residual HCl. Finally, the product was dried overnight in a vacuum oven for further characterizations including NMR analysis.^41^

### 2.2 Synthesis of Hf-EDB

Hf-EDB was synthesized based on our previously reported protocol for achieving nanoscale Hf-PEB but modifying the linker accordingly.^51^ Briefly, synthesizing Hf-EDB nanoparticles involves two steps: (1) preparation of the Hf precursor and the H_2_EDB solutions: Before the synthesis of Hf-EDB, stock solutions of HfCl_4_ and H_2_EDB were first prepared. The stock solution of HfCl_4_ was prepared by adding HfCl_4_ (2 mg/mL) to DMF and applying 30 min of sonication. The stock solution of H_2_EDB was prepared by adding H_2_EDB (2.5 mg/mL) to DMF and applying 10 min of sonication. To improve the dissolution of H_2_EDB, the H_2_EDB solution was heated for 20 min at 90 °C by using an oil bath. The resulting H_2_EDB solution was cooled and filtered with a 0.22 μm nylon syringe filter to remove the undissolved impurities. (2) Preparation of Hf-EDB nanoparticles using solvothermal reaction: To synthesize Hf-EDB, 50 mL of HfCl_4_ stock solution and 100 μL of TFA were added to a 150 mL vial followed by the addition of 50 mL of H_2_EDB stock solution. The mixture was sonicated for 10 min and heated for 72 h at 60 °C using the program-controlled furnace. After the reaction, the product was cooled down, centrifuged, and washed once with 10 mL of DMF and thrice with ethanol. Finally, the product was dried in a desiccator.

### 2.3 Incorporation of Pd NPs in Hf-EDB

The procedure was modified from our previous work.^41^ Hf-EDB (61 mg) was added to 5.0 mL of H_2_O and sonicated for 4 min using a probe sonicator. Then, 2.5 mL of H_2_O containing K_2_PdCl_4_ (42.75 mg) was added to the Hf-EDB suspension. The mixture was stirred for 30 min at room temperature. The resulting brownish particles (i.e., Pd^2+^@Hf-EDB) were collected by centrifugation (15000 rpm, 15 min) and rinsed 2 times with 5 mL of H_2_O (each time the mixture was sonicated for 10 min). Then, the particles were resuspended in 5.0 mL of H_2_O, and 2.5 mL of ice-cold water containing NaBH_4_ (49.55 mg) was added. The mixture was stirred for 30 min at room temperature. Finally, the blackish product was centrifuged and rinsed twice with 5 mL of H_2_O and once with 5 mL of EtOH before drying in vacuum. The overall preparation procedure of the Pd@Hf-EDB is summarized in Scheme 1.

**Scheme 1:**
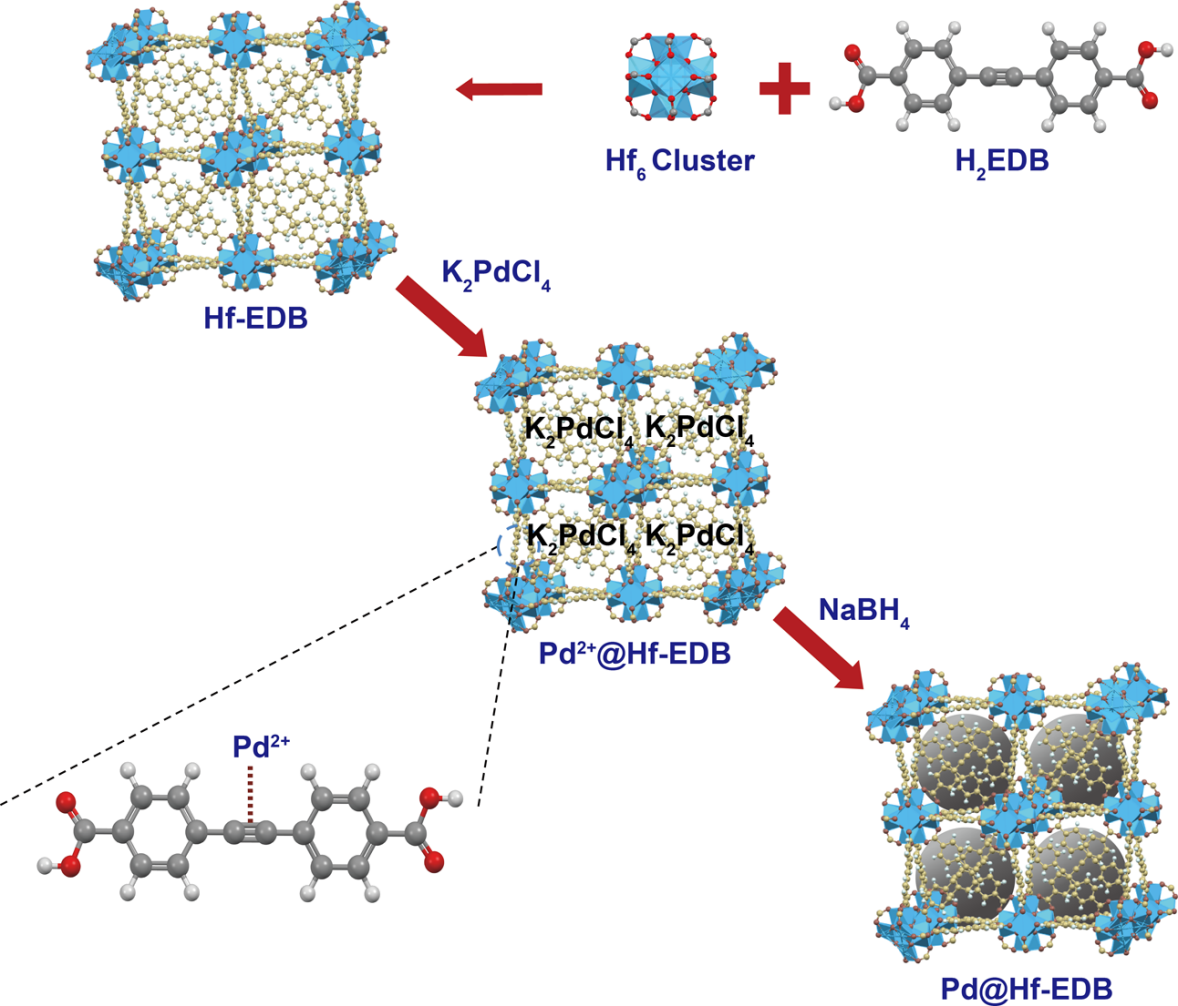
Synthesis of the Pd@Hf-EDB

### 2.4 Cell Viability

The cytotoxicity of materials was determined with an AlamarBlue assay (Figure S2). Typically, 2 × 10^4^ of BxPC-3 or RAW 264.7 cells in 100 μL of complete RPMI or DMEM medium was added to a well of a 96-well culture plate and incubated in a 37 °C, 5% CO_2_ incubator. The culture medium was replaced on the second day with 100 μL of complete RPMI or DMEM medium containing Hf-EDB and Pd@Hf-EDB (0–250 μg/mL). The cells were incubated for 24 h. And 20 μL of phosphate buffered saline (PBS) containing resazurin sodium salt (0.15 mg/mL) was added to the well. After 2 h of incubation, the fluorescence intensities (FI, λ_Ex_/λ_Em_ = 560/590 nm) of the samples were measured using an SpectraMax iD3Multi-Mode Detection Platform (Molecular Devices), and the background readings were subtracted using the wells containing culture medium, nanomaterial, and AlamarBlue reagent but without cells (Figure S3). The relative viabilities of the cells were then calculated using the equation: relative viability (%) = FI_Sample_/FI_Control_ × 100, where FI_Sample_ is the FI of the sample, and FI_Control_ is the mean FI of the control (0 μg/mL) group.

### 2.5 Observing the Cellular Uptake of Pd@Hf-EDB Using SXT

BxPC-3 cells were cultured on carbon-coated gold grids for the soft X-ray tomography (SXT) experiments. To improve the cell adhesion, the carbon-coated sides of gold grids were glow-discharged (15 mA, 25 s) using a PELCO easiGlow system from Ted Pella, Inc. (Redding, CA) to induce hydrophilicity. Subsequently, the prepared gold grids were positioned within a customized PDMS well. Then, 170 μL of BxPC-3 cell suspension (1 × 10^5^ cells/mL in complete RPMI) was added to each PDMS well. The cells were cultured overnight in a 37 °C 5% CO_2_ incubator. For the group treated with Pd@Hf-EDB, the culture medium was replaced with 170 μL of complete RPMI containing 100 μg/mL of Pd@Hf-EDB. The cells were then cultured for an additional 8 h in the same incubator. Following the incubation period, the cells were rinsed two times with PBS and stained with a staining solution containing Hoechst 33342 (4 μg/mL), Mitotracker Green FM (1 μM), and lysotracker Deep Red solution (1 μM) in serum-free RPMI medium at room temperature. The gold grid was rinsed with PBS, and then a 100 nm gold colloid solution (BBI Solutions, Crumlin, UK) was added as fiducial markers before the grid was plunged into liquid ethane using an EM GP plunge freezer from Leica (Vienna, Austria). The sample was kept in liquid nitrogen, and the locations of the cells on the grid were searched using Axio Imager A2 wide-field fluorescence microscopy from Zeiss. The fluorescence microscopy images of nuclei (Hoechst 33342), mitochondria (Mitotracker Green FM), and acidic compartments (lysotracker Deep Red) within the cells were also captured. After the screening, SXT was performed at the Taiwan Photon Source (TPS) 24A1 beamline at the National Synchrotron Radiation Research Center (NSRRC, Hsinchu, Taiwan).^52^ Flat-field correction of the SXT images was performed using customized software, and the 3D reconstruction of the tomogram was performed using IMOD software (https://bio3d.colorado.edu/imod/). The cryo-fluorescence and X-ray microscopy images (using the tilt series image at 0°) were correlated using Fiji software and Inkscape software (https://inkscape.org/). The channels were aligned according to the nucleus.

### 2.6 In Vitro CT Imaging

For in vitro CT imaging, agarose powder was dissolved in DI water at 65 °C to obtain a 2% agarose solution. Separately, imaging materials (Hf-EDB, iohexol, and Pd@Hf-EDB) were individually dispersed in DI water at 40 mg/mL using a probe sonicator (Q700, Qsonica LLC, Newtown, CT, USA) equipped with a probe (CL-334) at an intensity setting of 10 for 4 min. Subsequently, equal volumes of the heated 2% agarose solution and each sonicated dispersion were thoroughly mixed to yield stock solutions containing 1% agarose and 20 mg/mL of the imaging materials. Further serial dilutions were performed by mixing these stock solutions with a 1% agarose solution, achieving final material concentrations of 10, 5, and 2.5 mg/mL. Fractions of 100 μL from each diluted concentration were then transferred into 1.5 mL Eppendorf tubes and stored in a refrigerator. CT scans were conducted using a PET/SPECT/CT Tri-Modality Imaging System (GAMMA MEDICA-IDEAS, FLEX Triumph) with an X-ray tube voltage of 50 kVp. We first scanned the background, defining the grayscale value of air as −1000 and 2% agarose as 0. Subsequently, we scanned samples at different concentrations. Using Dragonfly software, we integrated the grayscale values of the samples and calculated the average. This mean gray value was then used in the formula Hounsfield unit (HU) = 1000 × (MGV_sample_ – MGV_agarose_)/(MGV_agarose_ – MGV_air_) to determine the HU values corresponding to each concentration. The relationship between the HU values and concentration was linear as the concentration increased.

### 2.7 In Vitro PA Imaging

For in vitro PA imaging, we dispersed Pd@Hf-EDB and GNRs in DI water. Pd@Hf-EDB was prepared at concentrations of 200, 100, 50, and 25 ppm, each with a volume of 20 mL. GNRs were prepared based on the peak optical density (OD) values, with OD = 1, 0.5, 0.25, 0.125, and 0.0625 in 20 mL volumes. The samples were then placed in 100 × 20 mm cell culture dishes. PA scanning was conducted by using a PAI system (FUJIFILM VisualSonics Inc. Vevo LAZR system). We applied a gel to the concave part of the PA ultrasound probe to serve as a medium for sound transmission, preventing noise from air interference. The probe was then placed on the surface of the samples, and scanning was performed at wavelengths ranging from 680 to 950 nm, with a scanning resolution of 10 nm. Five PA measurements at each wavelength were averaged using Vevo LAB software and plotted these averages against the corresponding concentrations.

To simulate the vascular environment, Pd@Hf-EDB and GNRs were dispersed in solutions at a concentration of 100 ppm, and each mixture was then injected seperately into polyethylene tubing with an inner diameter of 0.38 mm using insulin syringes. The concentration of GNRs was determined using ICP-OES. And, PA scanning was conducted at 750, 800, 850, 900, 950, and 975 nm using a custom-made dual-mode US/PA imaging system from the NHRI Liao lab. The integral image values of PA signals were extracted using MATLAB to represent the characteristic PA signals for each condition.

### 2.8 Animal Model

All animal experiments were carried out according to guidelines accepted by the National Health Research Institutes Laboratory Animal Center. This animal model study involves four male CAnN.Cg-Foxn1nu/CrlNarl mice, approximately 7 weeks old, that were born on October 7, 2024, and received from the National Laboratory Animal Center (NLAC) in Tainan, Taiwan, on November 15, 2024. The experiment was conducted on November 27, 2024, with the assigned protocol number of NHRI-IACUC-113079-M1. Briefly, to establish the xenograft tumor model, 10^6^ BxPC-3 cells were suspended in 50 μL of serum-free medium and mixed with 25 μL of Geltrex, then injected subcutaneously into the right thigh of the mice using a 0.5 mL 28G insulin syringe from BD Medical—Diabetes Care. The body weight of the mice ranged from 19 to 24 g. Xenografted tumor size was measured weekly in 2 orthogonal directions using calipers, and the tumor volume (mm^3^) was estimated using the equation: length × (width)^2^ × 0.5. The mice were sacrificed at 7 weeks after administration of the contrast agents.^53^

## 3 Results and Discussion

### 3.1 Synthesis and Characterization of H_2_EDB Linker

The H_2_EDB was synthesized using Sonogashira Cross-Coupling with methyl 4-iodobenzoate and methyl 4-ethynylbenzoate. Sonogashira cross-coupling forms carbon–carbon bonds between an alkyne and an aryl or vinyl halide. It typically involves the use of a palladium catalyst and a copper cocatalyst. An amine base environment deprotonates the alkyne, making it more nucleophilic. We synthesized the linker in-house considering commercial H_2_EDB linkers are challenging to acquire and often contain impurities. Our process for Me_2_EDB utilized nitrogen gas to reduce side reactions, while dichloromethane was employed to remove colored impurities. The NMR spectrum of Me_2_EDB in Figure 1a showed that the peak areas in CDCl_3_ matched the expected hydrogen ratios, indicating successful synthesis of Me_2_EDB. Additionally, the absence of any unidentified peak except minor peaks from residual solvent at 7.26 ppm and water at 1.56 ppm in CDCl_3_ solvent indicated that Me_2_EDB is of relatively high purity. Next, Me_2_EDB was hydrolyzed to H_2_EDB salts with KOH and converted to H_2_EDB using HCl. The NMR spectrum of H_2_EDB in Figure 1b showed that the peak areas matched the expected hydrogen ratios, and H_2_EDB is relatively pure, with only minor peaks from residual solvent at 2.5 ppm and water at 3.33 ppm in DMSO-*d*_6_ solvent. The carboxyl peak in the NMR spectrum was usually weak and broad, because the proton is involved in the rapid proton-exchange effect. This exchange occurs with the solvent or other carboxyl groups, causing the signal to become averaged and more diffused. Therefore, we also showed the ^13^C spectrum of the H_2_EDB in Figure S4, which matched the structure of H_2_EDB without an unidentified peak. Overall, the NMR results confirmed that H_2_EDB linkers have been successfully synthesized.

**Figure 1:**
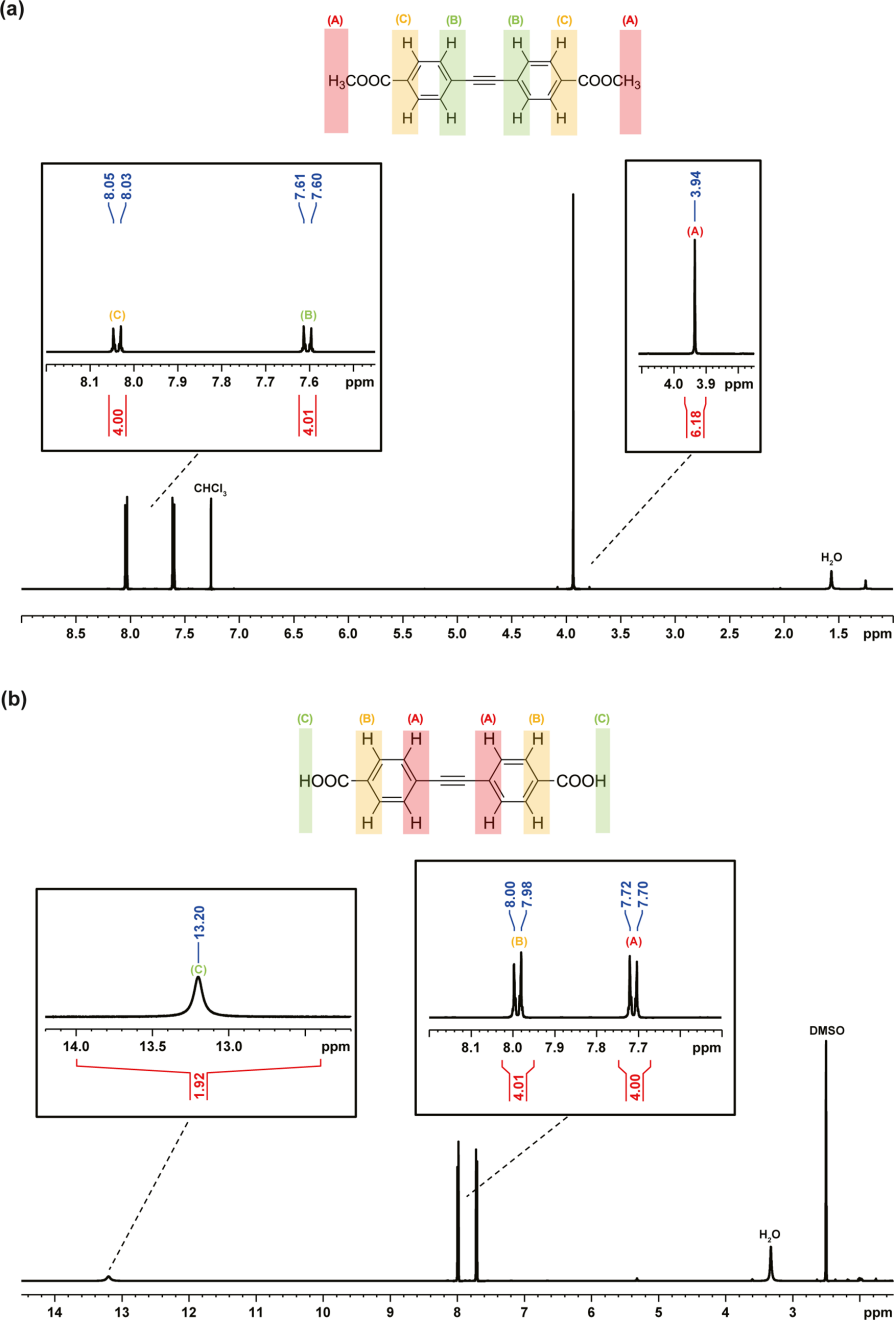
Nuclear magnetic resonance (NMR) spectra. (a) ^1^H NMR spectrum of Me_2_EDB, with CDCl_3_ as the solvent, (b) ^1^H NMR spectrum of H_2_EDB, with DMSO-*d*_6_ used as the solvent.

### 3.2 Synthesis and Characterization of Hf-EDB Nanoparticles

Previously, we had reported that nanoscale Hf-PEB can be synthesized by utilizing trifluoroacetic acid as the modulator through the solvothermal method.^54^ In this work, we changed the linker from H_2_PEB to H_2_EDB and used a similar ratio of the reactants to form a UiO type Hf-EDB [Hf_6_O_4_(OH)_4_(EDB)_6_]_*n*_ MOFs.

The X-ray diffraction (XRD) pattern of as-synthesized Hf-EDB is shown in Figure 2a, which was in agreement with that of the simulated structure of Hf-EDB.^100^ The five sharp characteristic peaks of Hf-EDB corresponding to the lattice planes (1 1 1), (2 0 0), (2 2 0), (3 1 1), and (2 2 2) were identified, which indicated high crystallinity and the absence of unidentified peaks concluded the purity of the as-synthesized MOF.

**Figure 2:**
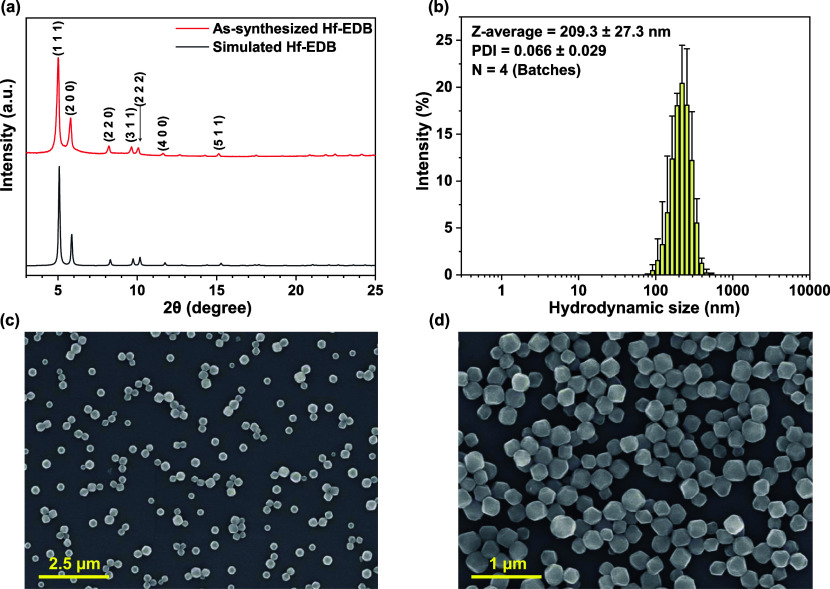
Characterization of Hf-EDB nanoparticles. (a) XRD patterns of Hf-EDB, (b) size distribution of Hf-EDB obtained from DLS measurements with four batches (*n* = 4), (c) SEM image of Hf-EDB with low magnification, (d) SEM image of Hf-EDB with high magnification.

Particle size and dispersity of the nanoparticles are critical factors in biomedical applications. Nanoparticles with a size of approximately 200 nm are more easily engulfed by cells.^55^ Therefore, during the synthesis of Hf-EDB nanoparticles, we utilized TFA as a modulator to regulate the particle size. We employed DLS analysis to uncover the particle size and dispersibility information. As shown in Figure 2b and Table S2, the size distribution of Hf-EDB had a *Z*-average of 209.3 ± 27.3 nm (*n* = 4) and a PDI of 0.066 ± 0.029 (*n* = 4). In the previous sentence, (*n* = 4) refers to four individual batches of Hf-EDB synthesized at different times. The small PDI indicated good dispersity, and the particle size of approximately 200 nm aligns well with our requirements for biomedical applications. We also measured the zeta potential of Hf-EDB to accertain its dispersibility. The results showed a zeta potential of approximately −37.9 ± 6.38 mV in PB buffer (pH 7.3), which indicated good dispersibility in a physiological environment (Table S3). We also measured the elemental composition of Hf-EDB using ICP-OES, and the results showed that Hf accounted for 37.60 ± 2.23%, similar to the molecular weight percent of Hf in Hf-EDB.

From the SEM images, we could observe that Hf-EDB aggregation is insignificant in the low-magnification image (Figure 2c). In the high-magnification image (Figure 2d), Hf-EDB nanoparticles exhibit well-defined particle outlines and a uniform size of approximately 200 nm, consistent with the results obtained from the DLS measurements.

### 3.3 Incorporation of Pd NPs and Characterization of Pd@Hf-EDB Nanoparticles

The ethynyl groups on the H_2_EDB linkers possess unique π-donor and π-acceptor properties that allows strong interactions with noble metal ions. The ethynyl groups within Hf-EDB initially absorbs the Pd^2+^ ions through stirring. Next, we used NaBH_4_ to reduce these absorbed Pd^2+^ ions into Pd NPs to attain Pd@Hf-EDB.

The SEM image of Pd@Hf-EDB nanoparticles (Figure 3a), showed well-defined particle outlines with some protrusions on the surface and a uniform size of approximately 200 nm. Further analysis using DLS was conducted to shed light on the observed particle size and dispersibility. As shown in Figure 3b, the size distribution of Pd@Hf-EDB had a *Z*-average of 265.1 ± 18.2 nm (*n* = 4) and a PDI of 0.183 ± 0.038 (*n* = 4). The results indicate that the Z-average and PDI of Pd@Hf-EDB are larger than that of Hf-EDB’s. This can be attributed to the fact that the synthesis of Pd@Hf-EDB is based on presynthesized Hf-EDB, meaning the state of Hf-EDB influences its characteristics. The presence of significant signals at the large particle size intensities in the size distribution due to aggregation and the introduction of Pd are inherently expected to bring about a slight increase in the *Z*-average. Despite these factors, the particle size and dispersibility of Pd@Hf-EDB are still suitable for biomedical applications. Additionally, the observed zeta potential of approximately −39.6 ± 7.58 mV in PB buffer (pH 7.3), indicated good dispersibility in a physiological environment. We also measured the elemental composition of Pd@Hf-EDB using ICP-OES, and the results revealed that Pd accounted for 25.74 ± 1.24% (Table S3).

**Figure 3:**
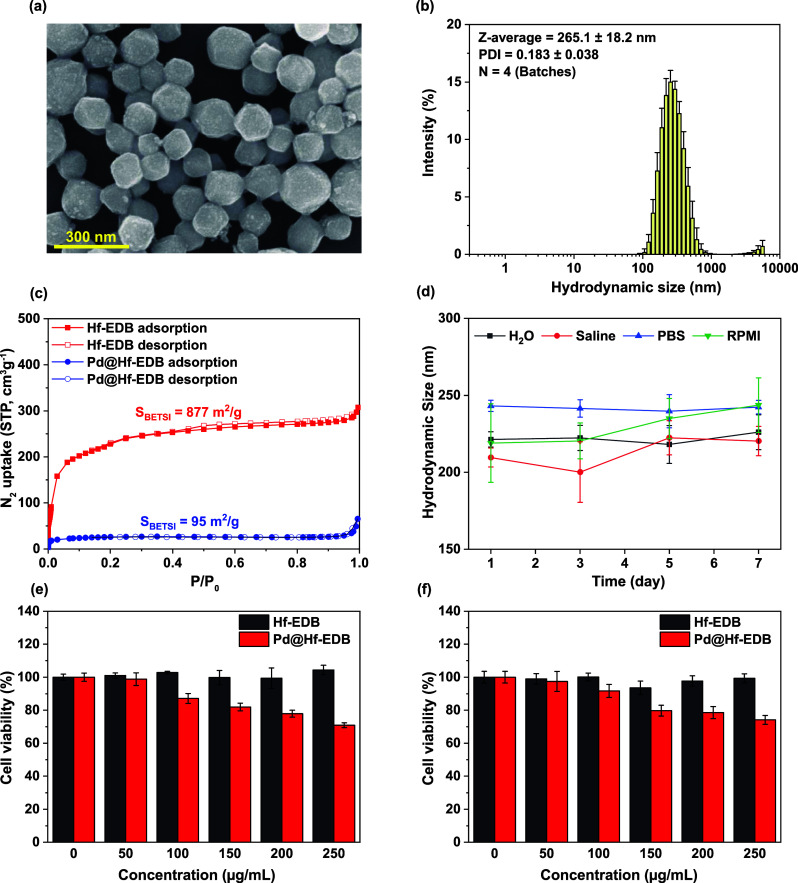
Characterization of Pd@Hf-EDB nanoparticles. (a) SEM image of Pd@Hf-EDB, (b) size distribution of Pd@Hf-EDB obtained from DLS measurements with four batches (*n* = 4), (c) N_2_ adsorption and desorption isotherms of Hf-EDB and Pd@Hf-EDB, (d) hydrodynamic size of Pd@Hf-EDB dispersed in different media at different storage time, (e) cell viability of BxPC-3 cells treated with Hf-EDB and Pd@Hf-EDB, (f) cell viability of RAW 264.7 cells treated with Hf-EDB and Pd@Hf-EDB.

To demonstrate the long-term stability of Pd@Hf-EDB as a contrast agent, we determined its hydrodynamic size using DLS over a period of 7 days (Figure 3d). The measurements were conducted in H_2_O, normal saline, PBS, and RPMI medium. The results clearly show that the contrast agent does not exhibit any significant changes in hydrodynamic size across these media for at least 7 days, indicating excellent stability. Additionally, to further assess the stability of Hf and Pd elements within the contrast agent, we monitored their leakage using ICP-OES analysis (Figure S7). Notably, after storage for 7 days, the released Pd was less than 0.5%, while Hf was nearly undetectable, demonstrating the contrast agent’s excellent chemical stability.

We also used nitrogen adsorption–desorption isotherms to observe the changes in the specific surface area before and after loading Pd NPs into Hf-EDB (Figure 3c). The results showed that the specific surface area decreased from 877 m^2^/g for Hf-EDB to 95 m^2^/g for Pd@Hf-EDB. This indicated that the successful loading of Pd caused a reduction in the specific surface area due to the filling of pores.

High-resolution transmission electron microscopy (HR-TEM) was performed to examine the morphology of Pd@Hf-EDB. The HR-TEM images (Figure 4a) clearly showed the spherical Pd NPs exhibiting distinct electron diffraction patterns. Furthermore, the HR-TEM in the inset in Figure 4a showed lattice fringes of 0.23 nm, which can be attributed to the (1 1 1) plane of the Pd crystal (Figure S5). The EDS mapping of Pd@Hf-EDB revealed that Hf and Pd were uniformly distributed throughout the particles (Figure 4b). Given that Hf signals are derived from Hf-EDB and Pd signals are derived from Pd NPs, we concluded that Pd NPs are successfully loaded into the pores of the Hf-EDB.

**Figure 4:**
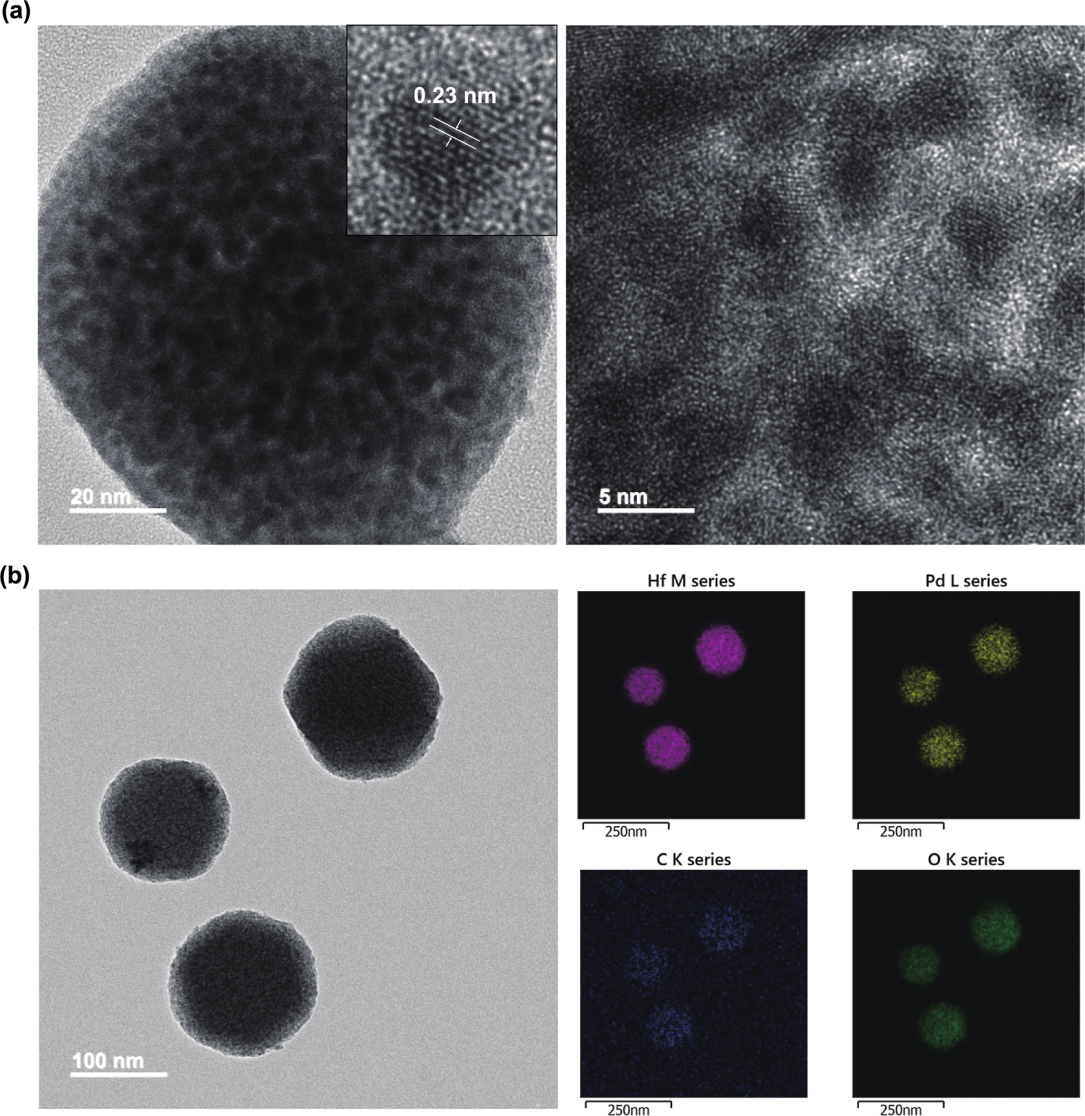
(a) HR-TEM image of Pd@Hf-EDB, (b) EDS mapping results of Pd@Hf-EDB.

The optical properties of the Pd@Hf-EDB nanoparticles were assessed through UV–vis spectral analysis (Figure S6a). The UV–vis spectrum revealed enhanced absorption in the 350–1100 nm range, which is attributed to Pd NPs’ strong interband absorption. This characteristic makes them suitable as PAI contrast agents in the NIR regions.

### 3.4 Cell Viability

Before conducting in vivo experiments, we investigated the materials’ toxicity to cells. As an imaging contrast agent for tumor cells, we want the materials to have no direct cytotoxicity to tumor cells. Therefore, we used the AlamarBlue assay to measure the cytotoxicity of the materials on tumor cells. We selected the BxPC-3 and RAW 264.7 cell lines as our cell models and cultured the materials at 0, 50, 100, 150, 200, and 250 μg/mL with the cells. Each well in the 96-well plate contained approximately 2 × 10^4^ cells. The results (Figure 3e,f) showed that Hf-EDB did not significantly reduce cell viability within this concentration range in both cell lines, while Pd@Hf-EDB maintained 85% cell viability in BxPC-3 and 90% cell viability in RAW 264.7 at 100 μg/mL. However, as the concentration continued to increase, there was a noticeable decline, with cell viability dropping to 70% in BxPC-3 and 75% in RAW 264.7 at 250 μg/mL. Therefore, we will use a 100 μg/mL concentration in subsequent imaging contrast agent efficacy experiments. Testing RAW 264.7 cells in addition to tumor cells is important to assess the cytotoxicity on macrophages, which can provide crucial insights into the Pd@Hf-EDB’s potential effects on the immune system.

### 3.5 Observing the Cellular Uptake of Pd@Hf-EDB Using SXT

To observe the cellular uptake of materials, we chose the NSRRC TPS 24A beamline: SXT. Compared to typical X-ray tomography, this beamline offers higher resolution while enabling visualization of organelles inside the cells via SXT. Due to the lower energy of soft X-rays, the organelles maintain a certain level of contrast. Using tomography and 3D reconstruction techniques, we can obtain three-dimensional structural images of the cell, such as the materials’ distribution. The ability to observe the behavior of the contrast agents within cells using SXT aligns perfectly with our end goal of developing CT imaging contrast agents. Compared to traditional CT, this technique allows us to observe at a more microscopic scale, revealing the three-dimensional distribution of the contrast agents after cellular uptake.

Figures 5a and S8 and S9 shows the images of BxPC-3 cells incubated with Pd@Hf-EDB under SXT at 0° and cryo-fluorescence microscopy. Figure S10 shows the images of BxPC-3 cells without incubation with Pd@Hf-EDB under SXT at 0° and cryo-fluorescence microscopy. We used cryo-fluorescence microscopy to observe the organelles of cells frozen in liquid nitrogen. The red channel showed the acidic compartments stained with LysoTracker, the blue represented the nucleus stained with Hoechst 33342, and the green indicated the mitochondria stained with MitoTracker. Pd@Hf-EDB was phagocytosed by the cells in significant quantities. The dense yet evenly distributed Pd@Hf-EDB with clearly visible outlines in the BxPC-3 cytoplasm (Figure 5a) implied that BxPC-3 cells had a strong affinity to engulf these nanoparticles. To further observe the distribution of Pd@Hf-EDB within the cell, we performed a three-dimensional cell reconstruction, as shown in Figure 5a. We chose this cell as it accumulated three distinct Pd@Hf-EDB aggregates, making it ideal for observation. Moreover, to confirm that these aggregates are inside the cell, we performed 3D reconstruction using SXT images from various angles and then divided the 3D image into 200 layers (Figure 5b).

**Figure 5:**
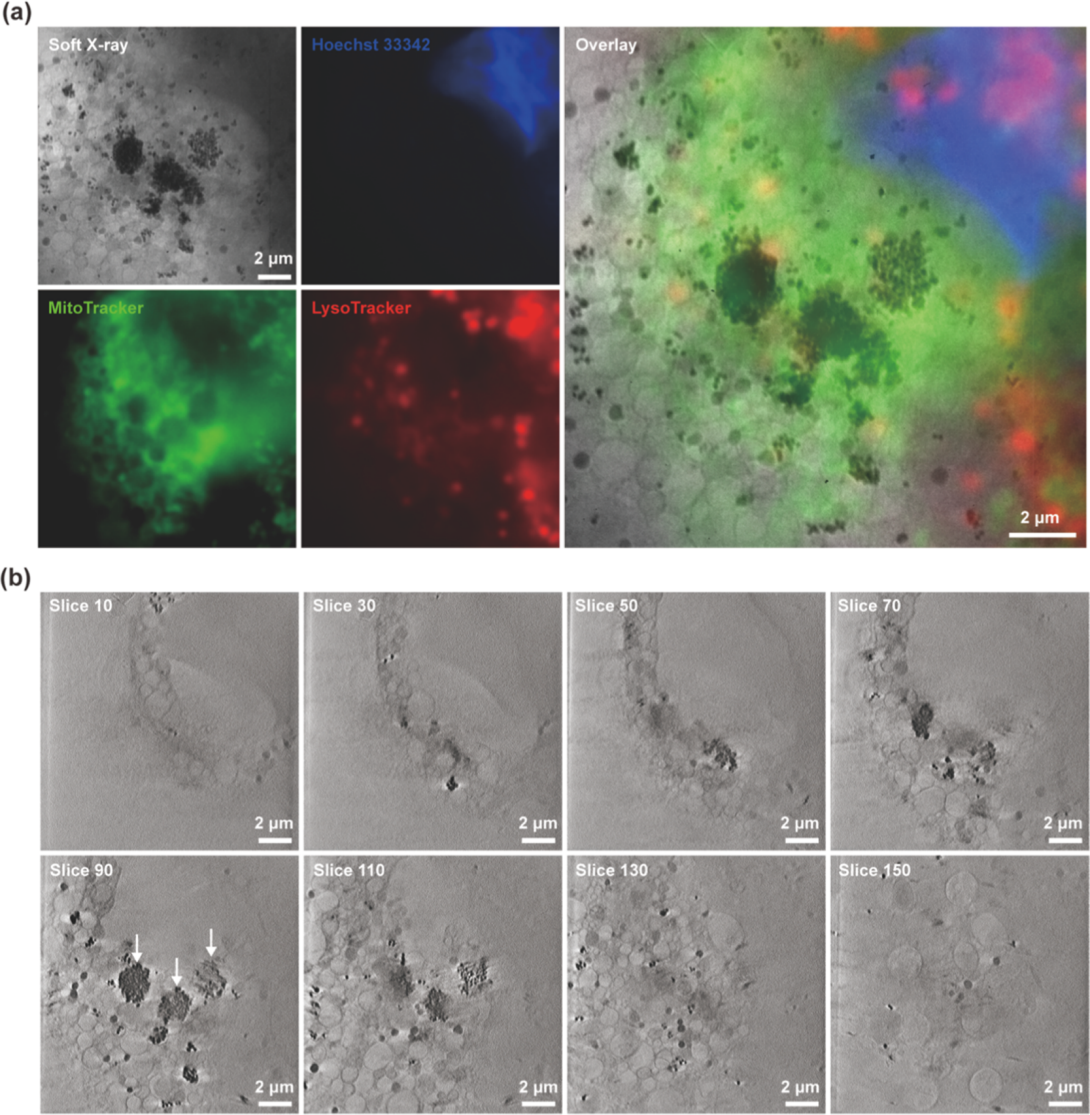
Cellular Uptake of Pd@Hf-EDB in SXT (a) SXT and cryo-fluorescence microscopy of the BxPC-3 cells phagocytosed Pd@Hf-EDB. The cell nucleus, mitochondria, and acidic compartment are stained by Hoechst 33342, MitoTracker, and LysoTracker, respectively. (b) The Z-stack SXT image was reconstructed into 200 slices. Arrow: Pd@Hf-EDB.

In Figure 5b, layers were cut downward from the top of the gold grid, meaning the first layer is at the top, and the last layer is near the carbon film. In the 30th slice, organelles and some Pd@Hf-EDB particles can be seen. By the 70th slice, one of the Pd@Hf-EDB aggregates appears. In the 90th layer, it becomes clearer that these three aggregates are formed by many small Pd@Hf-EDB particles, accompanied by numerous dispersed Pd@Hf-EDB particles nearby. By the 110th layer, these three aggregates begin to disappear, and by the 150th layer, the holey structures of carbon film start to appear. These results indicated that the Pd@Hf-EDB aggregates were located within the cytoplasm, with many smaller aggregates and individual Pd@Hf-EDB particles dispersed nearby.

Through microscopic observation of BxPC-3 cells ingesting Pd@Hf-EDB nanoparticles, we found that Pd@Hf-EDB can be engulfed by BxPC-3 tumor cells. Many studies have mentioned that nanoparticles can accumulate in tumor cells through the enhanced permeability and retention (EPR) effect. This effect presumably leads to a significant accumulation of Pd@Hf-EDB near tumors, making it an effective contrast agent.

### 3.6 In Vitro CT Imaging

We designed an in vitro experiment to understand the capabilities of Hf-EDB and Pd@Hf-EDB in enhancing X-ray CT imaging and to compare their performace with traditional molecular contrast agents such as iohexol. Wherein, Hf-EDB, Pd@Hf-EDB, and iohexol were separately dispersed in agarose gel at 0, 2.5, 5, 10, and 20 mg/mL concentrations to simulate a biological environment. Each mixture was then placed in an Eppendorf tube for analysis. Agarose was chosen due to its inert nature and ability to mimic the density and structure of biological tissues, providing physiologically relevant imaging results. Micro-CT imaging was performed to measure each sample’s HU values, quantitatively measuring the CT imaging capabilities. The HU values were calculated for each concentration using the formula HU = 1000 × (μ_sample_ – μ_agarose_)/(μ_agarose_ – μ_air_), the symbol μ represents the linear attenuation coefficient. This coefficient indicates how much a specific material attenuates X-ray radiation as it passes through. It quantifies the probability of photon interaction with the material, which is dictated by factors, such as density and atomic number. Higher μ values correspond to more excellent attenuation, leading to higher HU, used to assess the different contrast in medical imaging.^56^ In our calculation of HU, the μ value was determined using micro-CT scans. We set the reference values as follows: agarose gel was assigned a value of 0, and air was assigned a value of −1000. Additionally, we analyzed Hf-EDB, Pd@Hf-EDB, and iohexol at different concentrations dispersed within the agarose gel using Dragonfly software. By integrating their 3D images and calculating the average, we obtained the corresponding μ values for each group. Linear fitting of the CT value as a function of Hf-EDB, Pd@Hf-EDB, and iohexol concentrations in agarose gel were plotted to compare the imaging capabilities (Figure 6a). Phantom CT contrast images of Hf-EDB, Pd@Hf-EDB, and iohexol at different concentrations are shown in Figure 6b.

**Figure 6:**
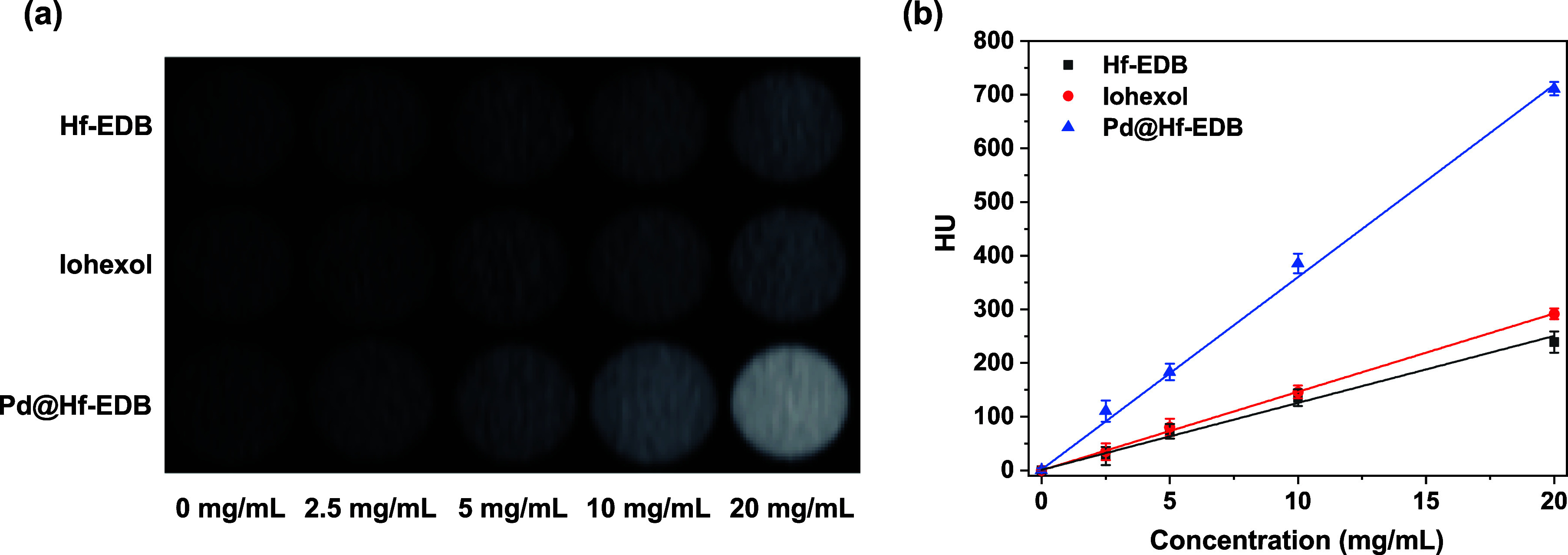
(a) In vitro phantom CT contrast images of Hf-EDB, iohexol, and Pd@Hf-EDB at different concentrations: 0, 2.5, 5, 10, 20 mg/mL. (b) CT values of Hf-EDB, iohexol, and Pd@Hf-EDB at different concentrations: 0, 2.5, 5, 10, and 20 mg/mL.

The results showed that Hf-EDB and iohexol exhibited similar CT imaging capabilities, as nearly overlapping attenuation curves indicated. This similarity could be attributed to hafnium’s high X-ray absorption properties (*Z* = 72) and iodine (*Z* = 53), which have high electron densities. In Hf-EDB, hafnium makes up about 38% by weight (from ICP-OES), while in iohexol, iodine accounts for approximately 46% by weight. These comparable weight percentages likely contributed to their comparable imaging performances. This similarity suggested that Hf-EDB, like iohexol, provided a baseline level of contrast enhancement suitable for many imaging applications. Furthermore, Pd@Hf-EDB demonstrated a significantly higher imaging capability. The attenuation curve for Pd@Hf-EDB was substantially above those of Hf-EDB and iohexol, with HU values approximately three times greater than those of iohexol at equivalent concentrations. The enhanced performance of Pd@Hf-EDB can be attributed to the presence of palladium, which may increase the X-ray attenuation properties because palladium (*Z* = 46) accounts for approximately 26% by weight in Pd@Hf-EDB. This increased attenuation is crucial towards improving the contrast in CT images, potentially allowing for more precise and detailed visualization of tissues.

While Iohexol often suffers rapid renal clearance and limited X-ray absorption capacity. Pd@Hf-EDB nanoparticles offer several advantages including enhanced accumulation in tumor tissues due to the EPR effect, which provides better contrast in cancer imaging,^57^ longer blood circulation times, allowing for prolonged imaging windows.^58^ Additionally, Pd@Hf-EDB can also be modified on the surface with targeting ligands to improve specificity for specific tissues or tumors, thereby highlighting the potential of this novel material, i.e., Pd@Hf-EDB nanoparticles as a promising candidate as CT contrast agent.

### 3.7 In Vitro PA Imaging

To understand the capacity of Pd@Hf-EDB nanoparticles as an efficient PA imaging contrast agent, we first investigated the Pd@Hf-EDB suspensions at various concentrations by ultraviolet–visible–near-infrared (UV–vis/NIR) absorption spectroscopy. The optical absorption spectra reveal a broad absorption band extending from 300 to 1300 nm (Figure S6b), with a particular focus on the NIR region beyond 700 nm (Figure 7a). Light in this range has a strong tissue penetration depth, making it suitable for medical applications such as photothermal therapy. Pd@Hf-EDB demonstrated a significant absorption level within this NIR region, which gradually decreased as the wavelength increased.^59^ For different concentrations (25, 50, 100, and 200 ppm), the normalized absorption intensity per characteristic cell length (*A*/*L*) is measured at specific wavelengths of 750, 800, 850, 900, 950, and 975 nm (Figure 7b). Applying the Lambert–Beer law, represented as *A*/*L* = α*C*, where *A* denotes absorbance intensity, *L* represents the cell length, α is the extinction coefficient, and *C* is the concentration, a linear relationship was observed between *A*/*L* and concentration, allowing for the calculation of extinction coefficients at the designated wavelengths (Table S1).

**Figure 7:**
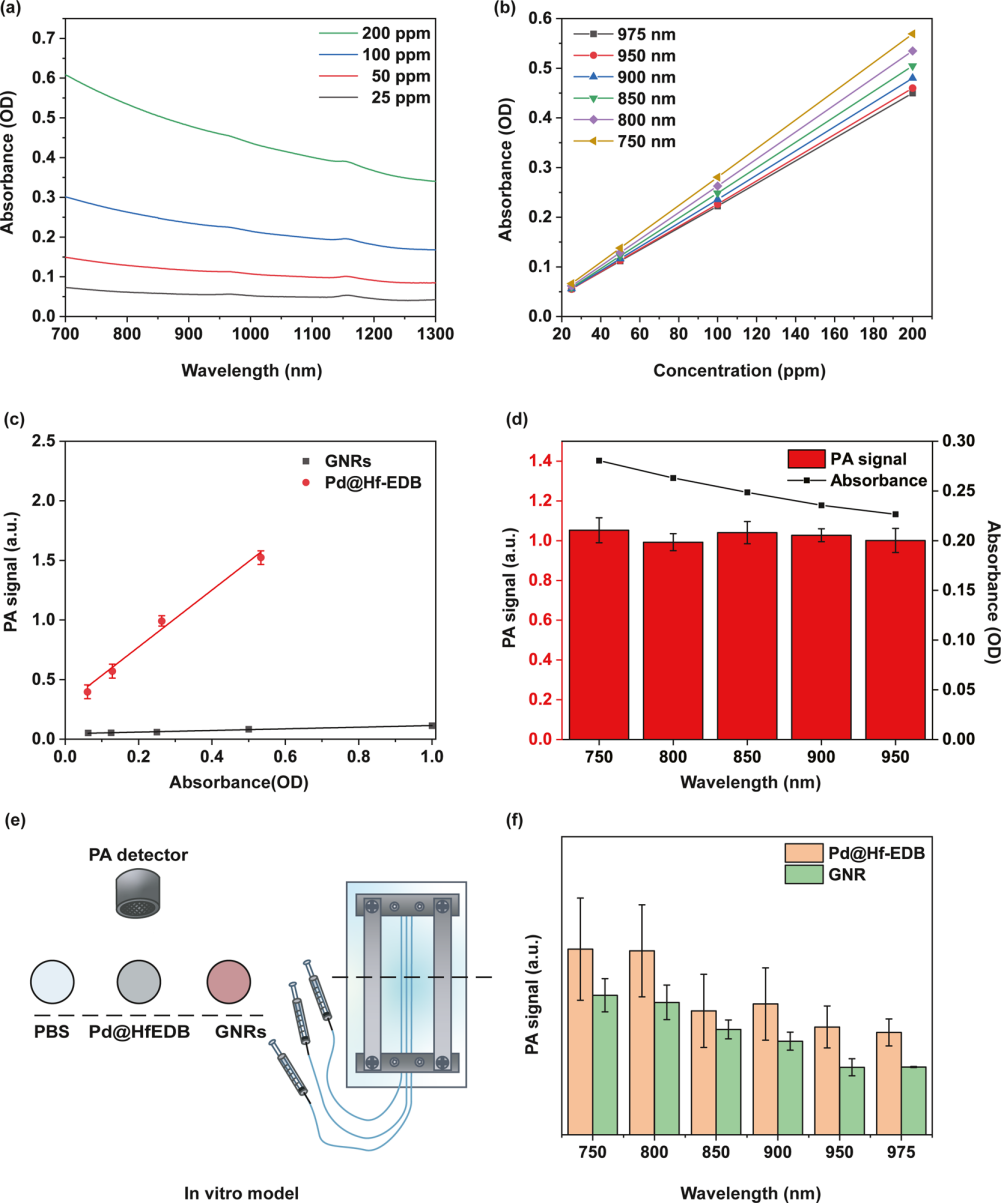
In vitro PA imaging of Pd@Hf-EDB nanoparticles. (a) Absorbance spectra of Pd@Hf-EDB dispersed in water at different concentrations. (b) The absorbance of Pd@Hf-EDB to varying concentrations for λ = 750, 800, 850, 900, 950, 975 nm. (c) PA signal of Pd@Hf-EDB and GNRs for different optical densities at 800 nm. (d) PA signal and absorbance of Pd@Hf-EDB at 100 ppm for different wavelengths. (e) In vitro model scheme for simulating blood vessels. (f) PA signal of Pd@Hf-EDB and GNRs at 100 ppm for different wavelengths.

To evaluate the PA performance of Pd@Hf-EDB compared to GNRs as PA contrast agents, we conducted an in vitro experiment. We dispersed varying concentrations of Pd@Hf-EDB and GNRs in 100 × 20 mm cell culture dishes namely 25, 50, 100, and 200 ppm. The OD values were calculated based on the calibration line shown in Figure 7b, while the OD for GNRs was obtained using UV–vis/NIR measurements. A coupling gel was applied underneath the ultrasound receiver and then attached to the liquid surface to measure the PA signals. To quantitatively compare the PA signal generation ability of Pd@Hf-EDB and GNRs, we analyzed the slopes of the PA signal versus OD values at 800 nm from Figure 7c. The slope for Pd@Hf-EDB was calculated to be 2.38, while for GNRs it was only 0.70, indicating that Pd@Hf-EDB exhibits a significantly stronger correlation between optical absorption and PA output signal. This suggests that Pd@Hf-EDB has a more enchanced conversion efficiency of absorbed light into acoustic energy. The higher slope can be attributed to the strong interband transition of Pd NPs in the NIR region and their enhanced photothermal stability, which helps retain consistent PA signal output under prolonged excitation without structural degradation. This suggests that Pd@Hf-EDB has superior PA contrast capabilities relative to GNRs, and the detection limit for Pd@Hf-EDB was determined to be as low as OD = 0.061 (equivalent to 25 ppm), highlighting its exceptional performance as a PA contrast agent. Additionally, Figure 7d illustrates the PA signals of Pd@Hf-EDB at 100 ppm across different wavelengths, revealing a trend similar to that observed in UV–vis/NIR measurements, with consistent PA signals in the NIR region. This correlation further reinforced the potential of Pd@Hf-EDB as an effective PA contrast agent, particularly in the NIR region, which is pivotal for deep tissue imaging.

Moreover, to simulate the vascular environment, Pd@Hf-EDB and GNRs were dispersed in solutions at a concentration of 100 ppm, and each mixture was then injected seperately into polyethylene tubing with an inner diameter of 0.38 mm using insulin syringes (Figure 7e). The PA signals were then measured using a custom-made Dual-mode US/PA imaging system from the NHRI Liao lab across the specified wavelengths of 750, 800, 850, 900, 950, and 975 nm. This wavelength range is selected based on the high tissue penetration of NIR light. The PA signals were analyzed, focusing on the longitudinal section of the simulated vascular model. The integrals of image value within the region of interest (ROI) were recorded as the PA signal for each material.

The results showed that under 800 nm laser irradiation, the PA signal intensity of Pd@Hf-EDB at 100 ppm was found to be 1.38 times higher than that of GNRs at the same concentration (Figure 7f). Additionally, PA signal trends observed for both Pd@Hf-EDB and GNR indicated a decrease with an increasing wavelength (Figure 7f). This trend aligns with the results obtained from UV–vis/NIR spectroscopy. When optically absorbing materials, namely, Pd@Hf-EDB and GNR are exposed to laser pulses shorter than the time required for thermal energy transport, they undergo transient thermoelastic expansion, producing a subsequent PA pressure wave. During this thermal expansion, the efficiency of converting light energy into a PA pressure wave is crucial for generating the PA signal. This conversion efficiency is primarily determined by the optical absorber’s light absorbance capabilities, photothermal conversion efficiency, and thermal properties such as heat capacity and thermal conductivity.^60^

The enhanced PA performance of Pd@Hf-EDB compared to GNRs can be attributed to two key factors: (i) the broader and stronger NIR absorption profile of Pd NPs, which leads to more efficient light-to-heat conversion, and (ii) the MOF framework of Pd@Hf-EDB, which may stabilize the embedded Pd NPs and minimize photothermal degradation or morphological deformation that GNRs commonly experience under prolonged laser exposure. These characteristics not only explain the superior PA signal output of Pd@Hf-EDB but also clarify why its PA signal trend closely follows the UV–vis/NIR absorbance spectrum. In contrast, the lower PA signals observed in GNRs presumable arises from scattering losses or instability during excitation. In conclusion, these findings support the idea that Pd@Hf-EDB is more effective and stable as a PA imaging agent in the NIR region, reinforcing its strong potential for biomedical imaging applications.

### 3.8 In Vivo PA Imaging

In this work, in vivo PA and CT imaging experiments were conducted using Pd@Hf-EDB as a CT/PA dual-modal contrast agent. We employed a xenograft tumor model in nude mice that was established by subcutaneously implanting BxPC-3 cancer cells. Once the tumor size reached approximately 150 mm^3^, we calculated the required amount of Pd@Hf-EDB based on the tumor size to achieve a concentration of 100 ppm. The compound was dispersed in 100 μL of PBS and injected subcutaneously near the tumor. About 30 min later, we used a PA instrument, initially utilizing ultrasound to locate the tumor. We then activated the laser to measure the PA signal and scanned a 3D image of the tumor area, approximately 1.5 cm in length, 1.3 cm in width, and 6 cm in depth. The length was divided into 20 sections to obtain cross-sectional views using a wavelength of 750 nm. The results demonstrated a strong PA signal contrast of Pd@Hf-EDB within the tumor, which overlapped with the tumor location in the ultrasound images (Figure 8a,b).

**Figure 8:**
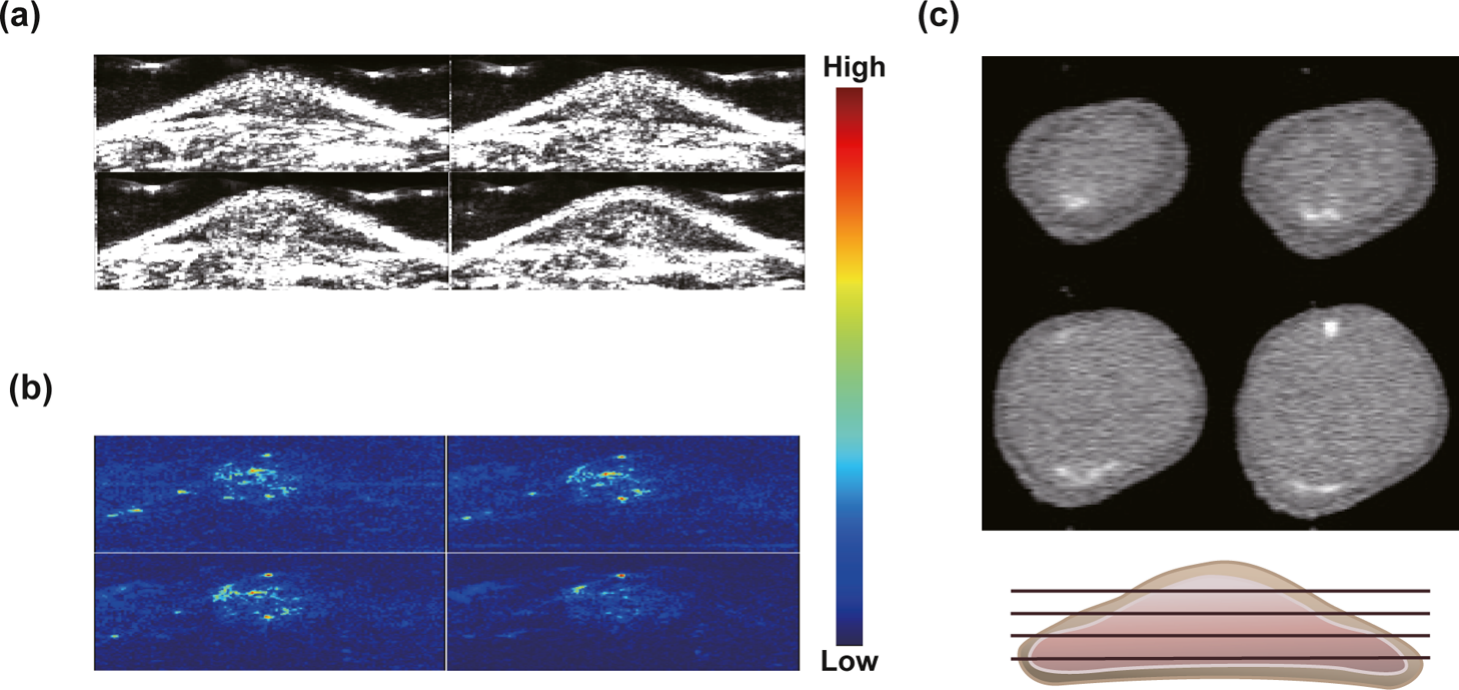
In vivo PA performance of Pd@Hf-EDB nanoparticles. (a) Ultrasound images of the BxPC-3 cells in the xenografted tumor in the nude mice. (b) A 3D sectional view of the PA images of the tumor at different positions postinjection of Pd@Hf-EDB. (c) A 3D sectional view of the CT images of the tumor at different positions postinjection of Pd@Hf-EDB.

After the PA experiment, we conducted the CT experiment using a micro-CT on the entire mouse body with a PET/SPECT/CT trimodality imaging system (GAMMA MEDICA-IDEAS, FLEX Triumph) at an X-ray tube voltage of 50 kVp. Using Fiji software, we examined the 3D cross-sectional images of the tumor (Figure 8c). The bright high-contrast spots represent our material Pd@Hf-EDB. Areas outside the bright spots did not show the obvious contrast of the material. We speculate that this observation is due to two reasons: first, the dosage used was based on cell viability tests and micro-CT requires a higher dosage for clear contrast. Thus, the bright spots may result from the aggregation of Pd@Hf-EDB due to uneven dispersion. Second, the energy of the micro-CT X-rays differs from the detectable energy range for metal particles, as it primarily aims for contrast in soft tissue. This is a common challenge for metal particle contrast agents, as different metals require specific X-ray energies.^43^

Although the subcutaneous injection did not necessarily facilitated uniform distribution of the contrast agent in the bloodstream, the results however showed a significant PA and CT signal. Combined with the EPR effect of the tumor region for nanoparticles, the contrast agent can accumulate more uniformly.^57^

## 4 Conclusions

In summary, we successfully synthesized Hf-EDB and loaded Pd NPs inside the pores of Hf-EDB to attain Pd@Hf-EDB nanoparticles. The biocompatibility of Pd@Hf-EDB was confirmed, maintaining 85% and 90% cell viability in BxPC-3 and RAW264.7 cells, respectively, at 100 μg/mL. Through SXT images of BxPC-3 cells ingesting Pd@Hf-EDB nanoparticles, we demonstrated that Pd@Hf-EDB can be readily engulfed by BxPC-3 tumor cells. A comparison with iohexol for in vitro CT imaging revealed that Hf-EDB possesses similar CT imaging capabilities, which can be attributed to its high hafnium content. However, Pd@Hf-EDB exhibited superior imaging performance, with attenuation values approximately three times greater than those of iohexol due to its significant palladium content enhancing X-ray attenuation. For in vitro PA imaging, Pd@Hf-EDB outperformed GNRs, showing a higher efficacy in the NIR region. This efficiency is improved by Pd@Hf-EDB’s structural stability and absorption properties, making it more effective than GNR, which may suffer from degradation or scattering under prolonged excitation. The in vivo results demonstrated strong PA and CT signal contrast of our material within the tumor, which overlapped with the tumor location in the ultrasound images. Overall, Pd@Hf-EDB possesses dual-modal capabilities, biocompatibility, and superior imaging performance, highlighting its potential for advanced imaging applications and offering more precise visualization and enhanced diagnostic accuracy.

## Supplementary Material



## Notes

Synthesis of Me_2_EDB via Sonogashira cross-coupling and hydrolysis of Me_2_EDB to form H_2_EDB; illustration of the AlamarBlue assay mechanism; experimental picture of the AlamarBlue assay; the NMR ^13^C spectrum of the H_2_EDB, with DMSO-*d*_6_ used as the solvent; lattice fringes of the Pd NPs inside the Hf-EDB; UV–vis absorption spectra of Hf-EDB and Pd@Hf-EDB; absorbance spectra of Pd@Hf-EDB at different concentrations; ICP-OES analysis showing residual Hf and Pd in supernatants after 7 days; SXT and cryo-fluorescence microscopy of BxPC-3 cells treated with Pd@Hf-EDB; additional SXT and cryo-fluorescence images of BxPC-3 cells treated with Pd@Hf-EDB; SXT and cryo-fluorescence microscopy of control BxPC-3 cells without Pd@Hf-EDB; extinction coefficients (α) of Pd@Hf-EDB at different NIR wavelengths; hydrodynamic size and PDI of Hf-EDB and Pd@Hf-EDB; and ICP-OES derived metal content and ζ-potential of Hf-EDB and Pd@Hf-EDB (DOCX)

## ∇

Taiwan Semiconductor Manufacturing Company, Zhunan Backend Fab, Miaoli, 35062, Taiwan
