# Supplementary material for: Development of Pd-Loaded Hf-Based Metal–Organic Framework as a Dual-Modal Contrast Agent for Photoacoustic Imaging and Computed Tomography
Source: ACS Biomater Sci Eng. 2025 May 6;11(6):3634–48. doi: 10.1021/acsbiomaterials.5c00169 (PMC12152835; doi:10.1021/acsbiomaterials.5c00169)
Supplement: Supplementary file 1 [file ab5c00169_si_001.docx]

**Supporting Information**

**Development of Pd-Loaded Hf-Based Metal-Organic Framework as a Dual-Modal Contrast Agent for Photoacoustic Imaging and Computed Tomography**

Yen-Chang Chen, #Yu-Sheng Yu, Yu-Kang Wang, R.K. Rakesh Kumar, Cho-yin Lee, Cheng-Hsin Chuang, Lun-De Liao, Kevin C.-W. Wu* (*Corresponding author)

**Affiliations:**

1. Department of Chemical Engineering, National Taiwan University, Taipei, Taiwan (Y Chen, Y Yu, Y Wang, K Wu)
2. Institute of Biomedical Engineering and Nanomedicine, National Health Research Institutes, Miaoli, Taiwan (Y Chen, Y Yu, R Kumar, L Liao, K Wu)
3. International Graduate Program of Molecular Science and Technology, Taiwan International Graduate Program, Academia Sinica, Taipei, Taiwan (K Wu)
4. International Graduate Program of Molecular Science and Technology (NTU-MST), National Taiwan University, Taipei, Taiwan (K Wu)
5. Department of Biomedical Engineering, National Yang Ming Chiao Tung University, Taipei, Taiwan (C Lee)
6. Department of Radiation Oncology, Taoyuan General Hospital, Ministry of Health and Welfare, Taoyuan, Taiwan (C Lee)
7. Institute of Medical Science and Technology, National Sun Yat-sen University, Kaohsiung, Taiwan (C Chuang)

**Author email addresses:**

Y Chen, na90724tw@gmail.com; Y Yu, f08524037@ntu.edu.tw; Y Wang, yvonne980032; R Kumar, rakesh141996@nhri.edu.tw; C Lee, choyin.lee@nycu.edu.tw; C Chuang, chchuang@imst.nsysu.edu.tw; L Liao, ldliao@nhri.edu.tw; K Wu, kevinwu@ntu.edu.tw.

**Experimental Section**

**Materials**

Ammonium chloride (NH_4_Cl, >99.5%), copper iodide (CuI, >98%), iohexol (>98.0%), methyl 4-iodobenzoate (>98%), sodium chloride (NaCl, >99.5%), and trifluoroacetic acid were purchased from Tokyo Chemical Industry Co., Ltd. (Tokyo, Japan). Bis(triphenylphosphine)palladium(II) dichloride (Pd(PPh_3_)_2_Cl_2_), Chloroform-d (99.8 atom%D), dichloromethane (DCM, 99%), dimethyl sulfoxide (DMSO), dimethyl sulfoxide-d_6_ (99.8 atom%D), gold(III) chloride trihydrate (>99.9%), gold standard for ICP (999 ± 2 mg/l), hafnium standard for ICP (999 ± 3 mg/l), hafnium chloride (HfCl_4_, 98%), HEPES solution (1M), hexadecyltrimethylammonium bromide (CTAB, >98%), palladium standard for ICP (999 ± 2 mg/l), potassium hydroxide (KOH), resazurin sodium salt, RPMI-1640 medium with L-glutamine, silver nitrate (AgNO_3_, >99%), sodium borohydride (NaBH_4_, >98%), sodium phosphate (Na_3_PO_4_, >96%), sodium bicarbonate (NaHCO_3_), sodium phosphate monobasic (NaH_2_PO_4_, >99%) and triethylamine (TEA, >99%) were purchased from Sigma-Aldrich (St. Louis, MO). Potassium tetrachloropalladate(II) (K_2_PdCl_4_, 98%) was purchased from Acros Organics (Geel, Belgium). N,N-dimethylformamide (DMF), tetrahydrofuran (THF, >99.9%) were purchased from DUKSAN (Ansan-si, Korea). Toluene (>99.5%) was purchased from Echo Chemical Co., Ltd. (Miaoli, Taiwan). Ethanol (EtOH, >99.8%), hydrochloric acid (HCl, ≥ 37%) and nitric acid (HNO_3_, ≥ 65%) were purchased from Honeywell (Charlotte, NC). 4-Ethynylbenzoic acid methyl ester (>97%) was purchased from Nova Materials Co., Ltd. (Taipei, Taiwan). Acetone (>99%), and hexane (>98.5%) were purchased from Union Chemical Works Ltd (Hsinchu, Taiwan). Ascorbic acid was purchased from Kanto Chemical Co., Inc (Tokyo, Japan). Agarose was from Bio-helix Co., Ltd. (New Taipei City, Taiwan). Antibiotic-antimycotic (Anti-anti) solution, fetal bovine serum (FBS), geltrex^TM^, and trypsin-EDTA (10X) were from Gibco (Grand Island, NY). Mitotracker Green FM was from MedChemExpress (Monmouth Junction, NJ). Hoechst 33342 and LysoTracker^TM^ deep red were purchased from Invitrogen (Carlsbad, CA). Quantifoil R2/2 circular holes on Au G200F1 finder grids (gold grids) was from Quantifoil Micro Tools GmbH (Großlöbichau, Germany).

**Synthesis of Gold Nanorods**

The protocol of GNR is from our previous work.^1^ The first step of preparing gold seed solution is as follows: 5 mL of 0.5 mM HAuCl_4_ was mixed with 5 mL of 0.2 M CTAB solution, and 0.6 mL of fresh 0.01 M NaBH_4_ was injected into the solution rapidly. The mixed solution was stirred for two hours, and the color of the mixed solution was changed from yellow to brownish-yellow. The second step was preparing the growth solution: 75 mL of 1 mM HAuCl_4_ was mixed with 75 mL of 0.2 M CTAB solution and 3.75 mL of 4 mM AgNO_3_ to control longitude absorption in the near-infrared region. 1.05 mL of 78mM ascorbic acid was added into the solution to reduce gold from Au^3+^ to Au^1+^. The solution color turned from yellow to colorless in seconds. Next, 180 μL of seed solution was added and kept in a 37 °C water bath for 24 h. The resulting solution was centrifuged at 20000 rpm for 10 min and washed thrice with water.

**Instrumentation**

**Scanning Electron Microscopy (SEM)**

For Hf-EDB and Pd@Hf-EDB samples, the powders were dispersed in ethanol and dropped onto silicon wafers. The silicon wafers with the samples were fixed onto the sample stub using conductive carbon tape. The SEM images were then acquired with an acceleration voltage of 10 kV using a cold-field emission scanning electron microscope (Hitachi S-4800) from Hitachi (Tokyo, Japan)

**X-ray Diffraction (XRD)**

X-ray diffraction patterns of the samples were measured using a Miniflex diffractometer from Rigaku (Tokyo, Japan), with Cu Kα radiation (λ = 1.5418 Å). Simulated patterns were generated using the crystallographic information files (CIF) of Hf-EDB retrieved from the CCDC 1062509 (<https://www.ccdc.cam.ac.uk>)^2-4^.

**Zeta Potential**

Hf-EDB (0.05 mg/mL) or Pd@Hf-EDB (0.05 mg/mL) were sonicated to disperse in a 10 mM phosphate buffer (6.5 mM NaH_2_PO_4_ + 3.5 mM Na_3_PO_4_, pH 7.32) and zeta potential was measured using a Zetasizer Nano instrument from Malvern Panalytical (Malvern, UK).

**Particle Size**

Hf-EDB or Pd@Hf-EDB disperse in ethanol or DI water via sonicated and measured using a Zetasizer Nano instrument from Malvern Panalytical (Malvern, UK).

**Specific Surface Area**

The N_2_ adsorption isotherms of Hf-EDB and Pd@Hf-EDB were measured with the ASAP 2020 N_2_ adsorption instrument from Micromeritics Instrument Corporation (Norcross, USA). The specific surface areas of the materials were determined using the recently reported Brunauer-Emmett-Teller Surface Identification (BETSI) method, using the BETSI v2.0 software retrieved from GitHub (https://github.com/fairen-group/betsi-gui).

**Nuclear Magnetic Resonance (NMR)**

To characterize the structure of linkers, the samples' nuclear magnetic resonance (NMR) spectra were obtained using an AVIII 500 MHz NMR spectrometer from Bruker (Billerica, MA). The data analysis was conducted using Bruker TopSpin Software.

**Transmission Electron Microscope (TEM)**

The distribution of the Pd NPs in Hf-EDB and the energy dispersive spectroscopy (EDS) mapping were observed using a JEM-2100F field-emission transmission electron microscope (FE-TEM) from JEOL Ltd. (Akishima, Japan).

**Inductively coupled plasma optical emission spectrometer (ICP-OES)**

The weight percent of Pd in Pd@Hf-EDB was obtained using an iCAP PRO inductively coupled plasma optical emission spectrometer (ICP-OES) from Thermo Fisher Scientific (Waltham, USA).

**Ultraviolet-visible (UV-Vis)**

Ultraviolet-visible (UV–Vis) spectra of the materials were obtained using a UV-Vis/NIR Spectrophotometer V-670 from JASCO (Tokyo, Japan).

**Cell line**

The human pancreatic cancer cell line BxPC-3 was obtained from the National Health Research Institute (NHRI) in Miaoli, Taiwan. The cells were grown in RPMI 1640 medium with L-glutamine supplemented with 1.5 g/L sodium bicarbonate, 10 mM HEPES, 10% FBS, and 1% anti-anti in a humidified atmosphere of 5% CO^2^ at 37 ℃.

**Cell subculture**

When the cells reached 80% confluence in the cell culture dish 100x20 well plate, the supernatant was removed, and the cells were rinsed with PBS. Then, 1 mL of trypsin was added, and the plate was placed in the incubator for 5 minutes. The cells were dispersed in 5 mL of complete RPMI medium and centrifuged at 1000 rpm for 5 minutes. They were then sub-cultured at a 1:3 ratio, adding 10 mL of complete medium before returning to the incubator.

**Dual-Modality US/PA Imaging System**

PA scanning was conducted using a custom-made Dual-mode US/PA imaging system from the NHRI Liao lab.^5-7^ The custom-made dark-field dual-modality ultrasound (US) and photoacoustic (PA) (i.e., US/PA) imaging system contained an 18.5-MHz high-frequency US transducer (L22-14 V, Verasonics, USA) and a customized light delivery system with fiber bundles. The received US/PA signals were associated with a multichannel high-frequency US platform (Vantage 128, Verasonics, USA). The laser excitation should be synchronized with the US information obtained for the PA mode imaging. A customized dark-field illumination system with fiber bundles was used to efficiently deliver the laser energy to the region of interest (ROI) and form a PA dark-field between the focus point and US transducer for a better signal-to-noise ratio (SNR). The PA imaging resolution was estimated based on the full width at half-maximum (fwhm) of each Gaussian function from the signals and was measured to be 124 ± 31 μm for the developed system. The in vitro and in vivo PA B-scans were analyzed using a custom-made interface based on MATLAB (R2007a, MathWorks, USA). The maximum permissible exposure was well within the American National Standards Institute (ANSI) (i.e., less than 20 mJ·cm−2) during the experiment.

**Comparative analysis with leading technologies**

In recent years, many research groups have begun to explore the application of MOFs.^8^ On the other hand, traditional inorganic nanocarriers such as iron oxide (Fe_3_O_4_) nanoparticles have been widely utilized in MRI diagnostics due to their good magnetic properties and biocompatibility.^9^ However, these nanoparticles often face challenges related to difficulties in surface modification,^10^ limited drug loading capacity, and issues in achieving precise drug release control.^11^ Additionally, gold nanoparticles, although exhibiting outstanding contrast performance in CT imaging, are limited in clinical application due to potential cellular toxicity at high dosages and insufficient biodegradability.^12^

In comparison, MOFs can efficiently load various imaging probes and allow facile tuning of physicochemical properties by selecting appropriate metal ions or organic linkers. Their easily functionalizable surfaces, particularly ion clusters capable of coordinating biomolecules, further enhance tumor-targeting specificity, which is a significant advantage over non-MOF materials.^8^

Many current studies on MOF-based contrast agents have focused primarily on MRI applications.^13^ This is largely attributable to the outstanding magnetic properties and easily tunable structures of MOFs, which significantly enhance MRI image contrast. For instance, Wang et al.^14^ introduced that Gd^3+^- or Mn^2+^-based MOFs have excellent performance as MRI contrast agents. In addition, some researches focus on MOFs as contrast agents for CT imaging; for example, K. E. deKrafft et al.^15^ developed Zr and Hf-based MOFs for use as contrast agents in CT imaging.

However, studies employing MOFs for dual-modal imaging applications are relatively scarce compared to conventional non-MOF materials. For example, Rieter et al.^16^ reported on nanoscale Gd^3+^-based MOFs as multimodal imaging contrast agent for combining MRI with optical imaging. Tian et al.^17^ integrated gold nanoparticles with Gd-based MOFs to develop a dual-modal contrast agent for CT and MRI imaging. In particular, these MOFs dual-modal contrast agents have rarely involved PAI. Compared to relatively common CT/MRI dual-modal imaging combinations, Pd@Hf-EDB as a CT/PA dual-modal contrast agent demonstrates several distinct benefits. Although MRI provides excellent soft-tissue contrast, it suffers from relatively low sensitivity and long scanning times.^18^ On the other hand, PAI combines the high molecular sensitivity of optical imaging with real-time monitoring of molecular and functional information within the tumor microenvironment,^19^ such as tumor angiogenesis,^20^ oxygenation, and hypoxic conditions,^6, 21^ which information that traditional MRI techniques usually cannot provide quickly and sensitively. Furthermore, combining CT and PAI provides effective complementary information with anatomical details in CT imaging. Thus, the combination of CT and PAI imaging technology is highly suitable for efficient tumor diagnosis and localization applications.

Overall, Pd@Hf-EDB combines the advantages of MOFs with the dual-modal imaging capabilities of CT and PAI, marking the first reported application of nanoscale Hf-based MOFs in these CT/PA dual-modal technologies.

**Study limitations**

In this study, we focused on the design and application of MOF-based nanomaterials and demonstrated the potential of Pd@Hf-EDB as a dual-modal contrast agent for CT and PAI. We have proved its imaging capabilities through preliminary subcutaneous injection models. However, in clinical application, contrast agents are typically administered intravenously. The MOF’s framework of Pd@Hf-EDB offers the advantage of easy surface modification; specifically, ion clusters on the framework can be utilized to coordinate targeting molecules containing carboxyl functional groups,^22^ thereby enhancing tumor specificity. When combined with cancer treatment, this approach also has the potential for theranostic applications.

However, before such applications can be realized, assessing the ethical considerations of using Pd@Hf-EDB as a contrast agent is crucial. Because Pd@Hf-EDB comprises metal components (Hf and Pd) and organic linkers (H_2_EDB), their long-term biological interactions and potential toxic effects remain insufficiently understood. The metabolism and excretion pathways of Pd@Hf-EDB are primarily influenced by its nanoparticulate nature. MOFs, such as Hf-EDB, typically undergo gradual degradation within physiological environments, releasing their metal components (Hf and Pd) over time. Hf as a high-Z element, generally exhibits low toxicity and limited bioavailability, consistent with existing literature on hafnium-based materials used clinically or in advanced biomedical research.^23^ However, Pd necessitates careful consideration due to its potential for toxicity upon prolonged exposure.^24^ Nanoparticles administered intravenously or subcutaneously are often subject to uptake by the reticuloendothelial system (RES), predominantly in organs such as the liver, spleen, and kidneys.^25^ Accumulation of Pd@Hf-EDB nanoparticles in these organs may lead to risks of toxicity if Pd ions are excessively released. Therefore, longitudinal studies investigating biodistribution, accumulation kinetics, and clearance mechanisms of Pd@Hf-EDB are essential for thorough safety evaluation. Future work should involve histological analysis and elemental quantification in vital organs at multiple time points post-administration to assess potential chronic effects.

Comparatively, clinically utilized contrast agents, such as iodine-based agents for CT and gadolinium-based agents for MRI, often exhibit rapid renal excretion and minimal long-term accumulation. However, these agents also present significant limitations, including nephrotoxicity and potential nephrogenic systemic fibrosis.^26, 27^ Pd@Hf-EDB nanoparticles, due to their size and surface properties, may offer a more extended viewing time and reduced acute toxicity compared to these traditional agents. Nevertheless, comprehensive toxin analysis is necessary to conclusively determine their relative safety.

In addition to patient safety, the environmental impacts associated with the synthesis, usage, and disposal of Pd@Hf-EDB must be carefully considered. As previously mentioned, the synthesis involves potentially hazardous solvents such as dimethylformamide (DMF), metal ions (Hf and Pd), and organic linkers (H_2_EDB). According to recent literature on MOFs, the presence of heavy metals and organic solvents used during synthesis can lead to significant environmental toxicity if not managed properly. For instance, solvents such as DMF are known to pose risks due to their toxicity and persistence in the environment.^28^ Additionally, metal ions such as Pd^2+^ and Hf^4+^ tend to be oxidized to form metal oxide, which may have toxic effects in our environment.^28^

Recent studies highlight the importance of adopting green synthesis techniques to mitigate these environmental concerns. Sustainable synthesis strategies involving water solvents, supercritical liquids, ionic liquids, or bioderived solvents have been proposed as safer alternatives to conventional hazardous solvents.^29^ Although the current synthesis of Pd@Hf-EDB requires the use of DMF, we can move towards developing alternative synthesis methods that replace DMF with safer and more sustainable solvents.

Furthermore, proper procedures for disposal, recycling, and regeneration processes such as solvent exchange, thermal activation, vacuum treatment, and supercritical CO₂ methods have been recommended to enhance sustainability in MOF usage.^28^ These methods can help minimize hazardous waste generation and reduce contamination risks associated with MOF disposal. Thus, it is essential for researchers involved with Pd@Hf-EDB to adopt sustainable synthesis methods and develop appropriate recycling and disposal strategies to minimize potential ecological harm and to promote environmentally friendly practices in MOF research and applications.

Figure S1. Synthesis of H_2_EDB. (a) Preparing Me_2_EDB through Sonogashira Cross-Coupling. (b) Hydrolysis of the Me_2_EDB to form H_2_EDB.

Figure S2. Mechanism of the AlamarBlue assay.


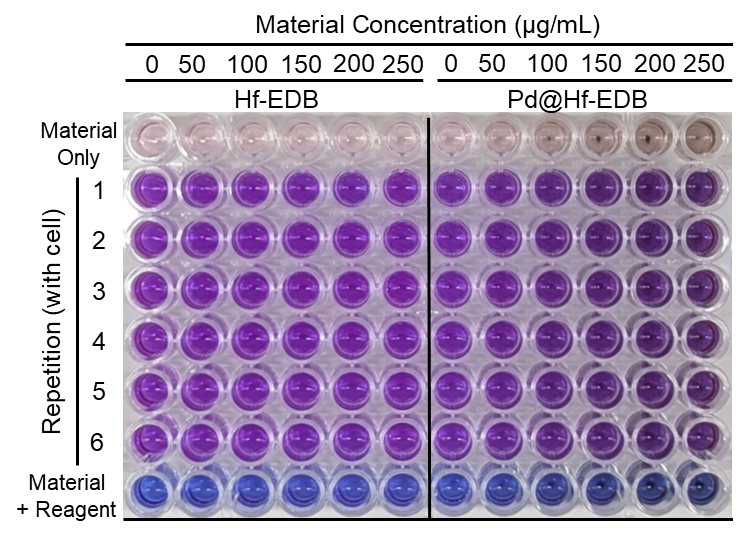


Figure S3. The detail of the AlamarBlue assay experiment.


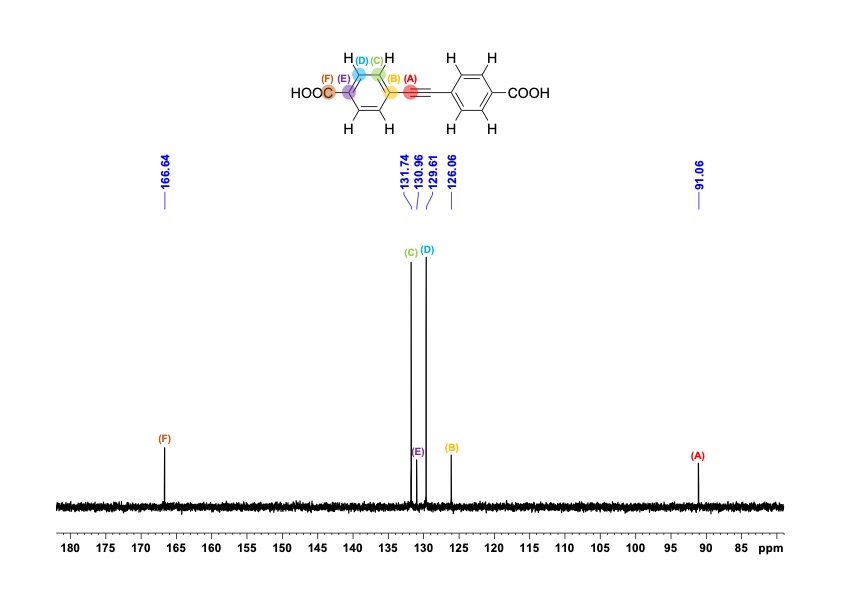


Figure S4. The NMR ^13^C spectrum of the H_2_EDB, with DMSO-*d*_6_ used as the solvent.

**Figure S5.** Lattice fringes of the Pd nanoparticles inside the Hf-EDB.

**Figure S6.** (a) The absorbance spectra of Hf-EDB and Pd@Hf-EDB. (b) The absorbance spectra of Pd@Hf-EDB dispersed in water at different concentrations.

**Figure S7.** Percentage of original Hf and Pd content remaining in the supernatants of Pd@Hf-EDB (1 mg/mL) after being stored at 37 °C for 7 days.


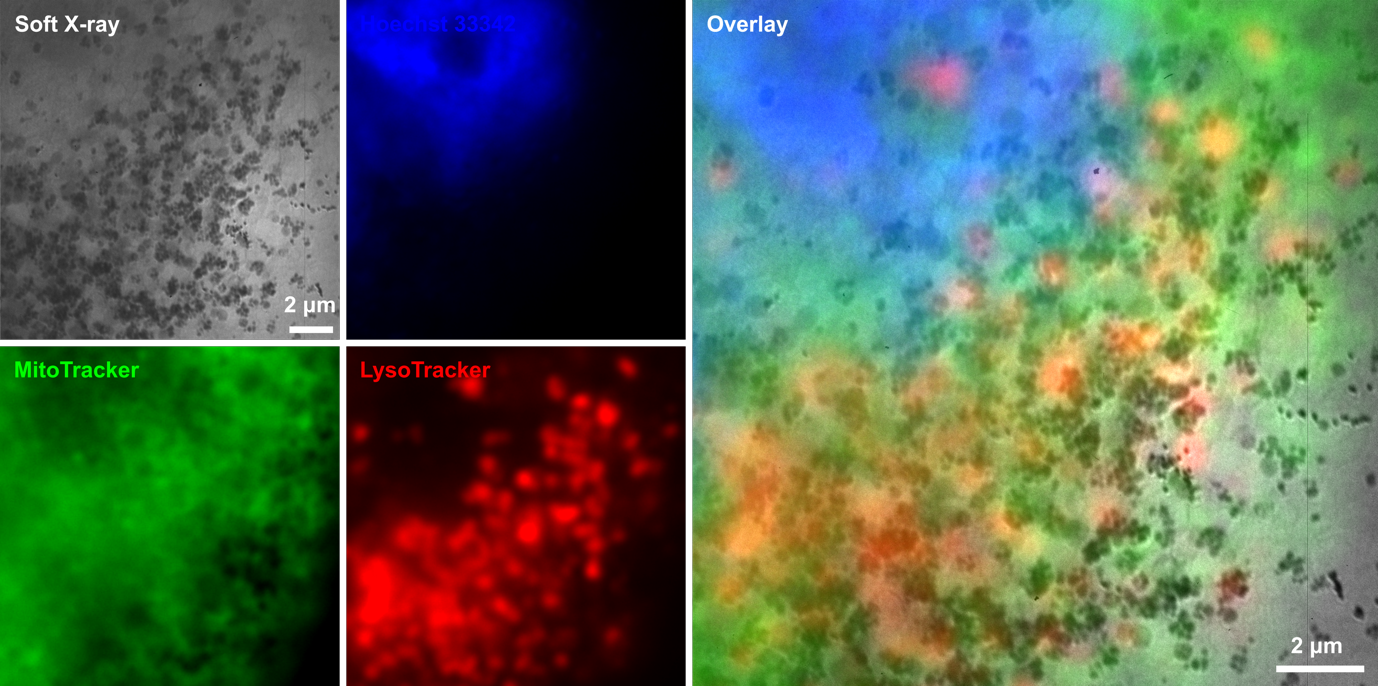


**Figure S8.** SXT and cryo-fluorescence microscopy of the BxPC-3 cells phagocytosed Pd@Hf-EDB.


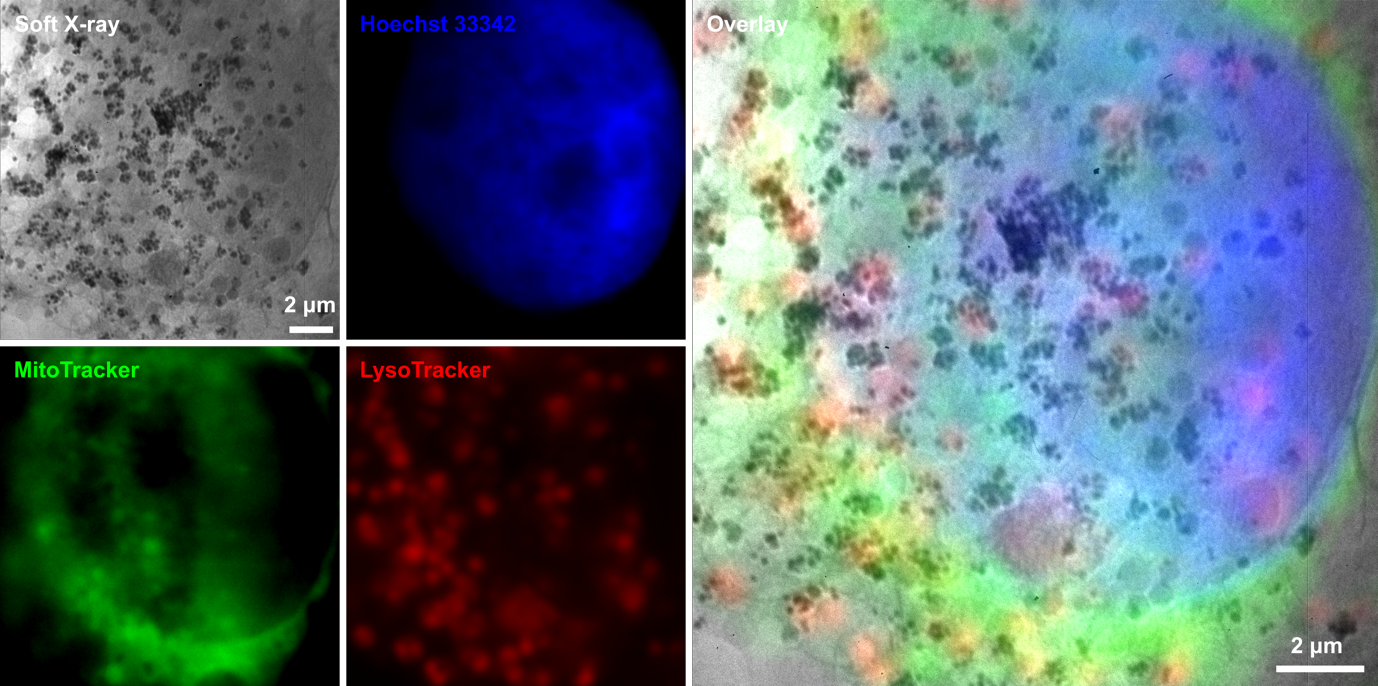


**Figure S9.** SXT and cryo-fluorescence microscopy of the BxPC-3 cells phagocytosed Pd@Hf-EDB.


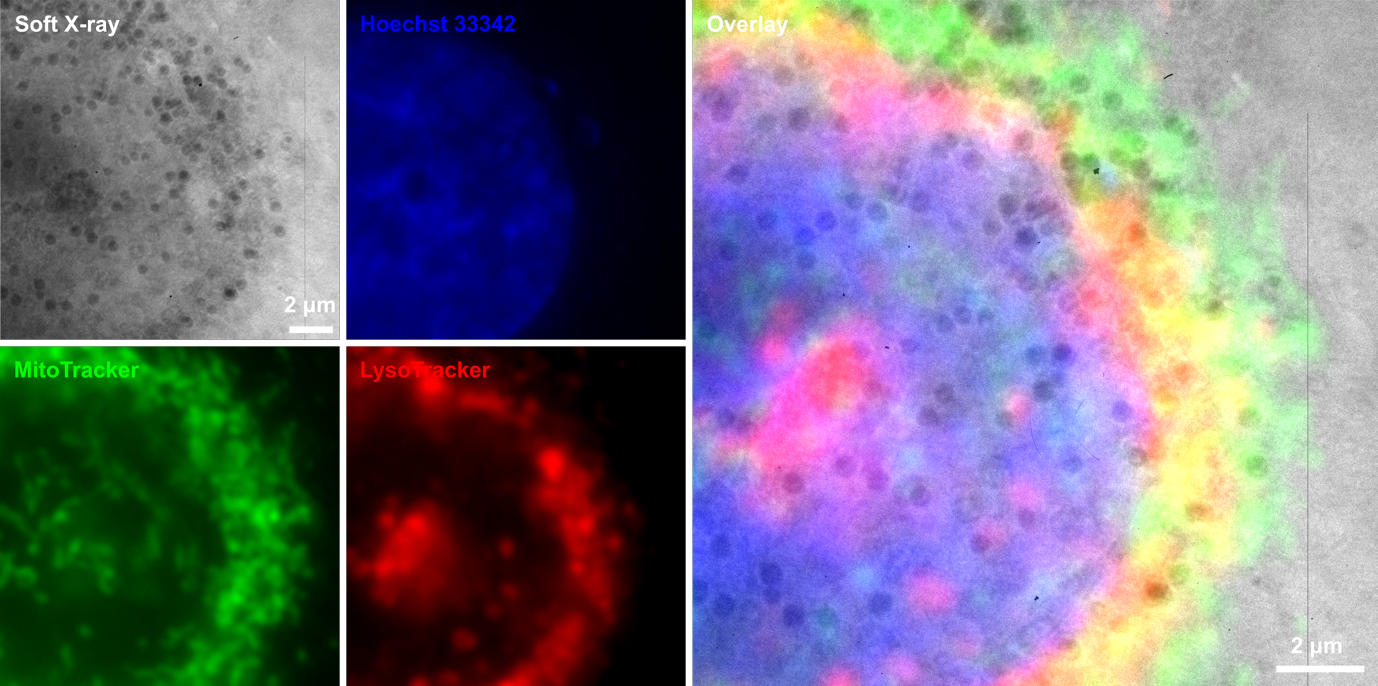


**Figure S10.** SXT and cryo-fluorescence microscopy of the BxPC-3 cells without Pd@Hf-EDB.

**Table S1.** Extinction coefficients at different wavelengths.

| **λ (nm)** | $\boldsymbol{\alpha}$ **(Lg^-1^cm^-1^)** |
| --- | --- |
| **750 nm** | **2.9** |
| **800 nm** | **2.7** |
| **850 nm** | **2.6** |
| **900 nm** | **2.4** |
| **950 nm** | **2.3** |
| **975 nm** | **2.3** |

**Table S2.** Hydrodynamic Size and PDI of Hf-EDB and Pd@Hf-EDB.

| **Materials** | **Hydrodynamic size (nm)** | **PDI** |
| --- | --- | --- |
| Hf-EDB | 209.3 ± 27.3 | 0.066 ± 0.029 |
| Pd@Hf-EDB | 265.1 ± 18.2 | 0.018 ± 0.038 |

**Table S3.** Metal weight percent and ζ-potential of Hf-EDB and Pd@Hf-EDB.

| **Materials** | **Metal wt%** | **ζ-potential (mV)** |
| --- | --- | --- |
| Hf-EDB | Hf: 37.60 ± 2.23 | -37.9 ± 6.38 |
| Pd@Hf-EDB | Pd: 25.74 ± 1.24 | -39.6 ± 7.58 |

**Table S4.** Comparative Analysis of Pd@Hf-EDB with Leading Imaging Technologies

| **Materials** | **MOF** | **Dual-modal** | **Imaging types** | **Advantages** | **Disadvantages** | **Ref.** |
| --- | --- | --- | --- | --- | --- | --- |
| Fe_3_O_4_ NPs | No | No | MRI | Good biocompatibility  Good magnetic properties | Limiting drug loading  Surface modification challenges | [9] |
| Gold NPs  Gd or Mn-based MOF  Zr or Hf-based MOF  Gd-based MOF  Gold NPs/Gd-based MOF | No  Yes  Yes  Yes  Yes | No  No  No  Yes  Yes | CT  MRI  CT  MRI/optical  CT/MRI | High CT contrast performance  Tunable structures  Good MRI contrast  Tunable structures  Good CT contrast  Dual-modality  Tunable structures  Dual-modality  Tunable structures | Limiting drug loading  Insufficient biodegradability  Single modality  High cost  Single modality  High cost  Low sensitivity  High cost  Low sensitivity  High cost | [10]  [14]  [15]  [16]  [17] |
| Pd@Hf-EDB | Yes | Yes | PA/CT | Dual-modality  High sensibility | Requires further investigations to ensure targeted binding | This work |

**Table S5.** Comparative Analysis of Pd@Hf-EDB with Other Dual-modal Imaging Materials

| **Materials** | **Imaging types** | **Advantages** | **Disadvantages** | **Ref.** |
| --- | --- | --- | --- | --- |
| DOTA–QD–RGD | PET/NIRF | High specificity  Low toxicity | Low in vivo targeting efficiency  Surface modification challenges | [30] |
| Gd/CuS@PEI-FA-PS NGs  GM-PET Fusion  Au-AgI core/shell  Gd-based MOF  Gold NPs/Gd-based MOF | MR/PA  MRI/PET  CT/PA  MRI/optical  CT/MRI | Effective photothermal performance  Excellent dispersion  Detailed structural information  Enhanced functional diagnosis  Effective dual-modal imaging  Good stability  High structural tunability  Tunable optical properties  High structural tunability  Good stability | Synthetic complexity  Unclear stability  Synthetic complexity  Unclear stability  Complex synthesis  Surface modification challenges  Low sensitivity  Synthetic limitations  Complex synthesis  High cost | [31]  [32]  [33]  [16]  [17] |
| Pd@Hf-EDB | PA/CT | High structural tunability  High sensitivity | High cost  Equipment limitations | This work |

**Institutional Animal Care and Use Committee (IACUC): Approval Certificate**


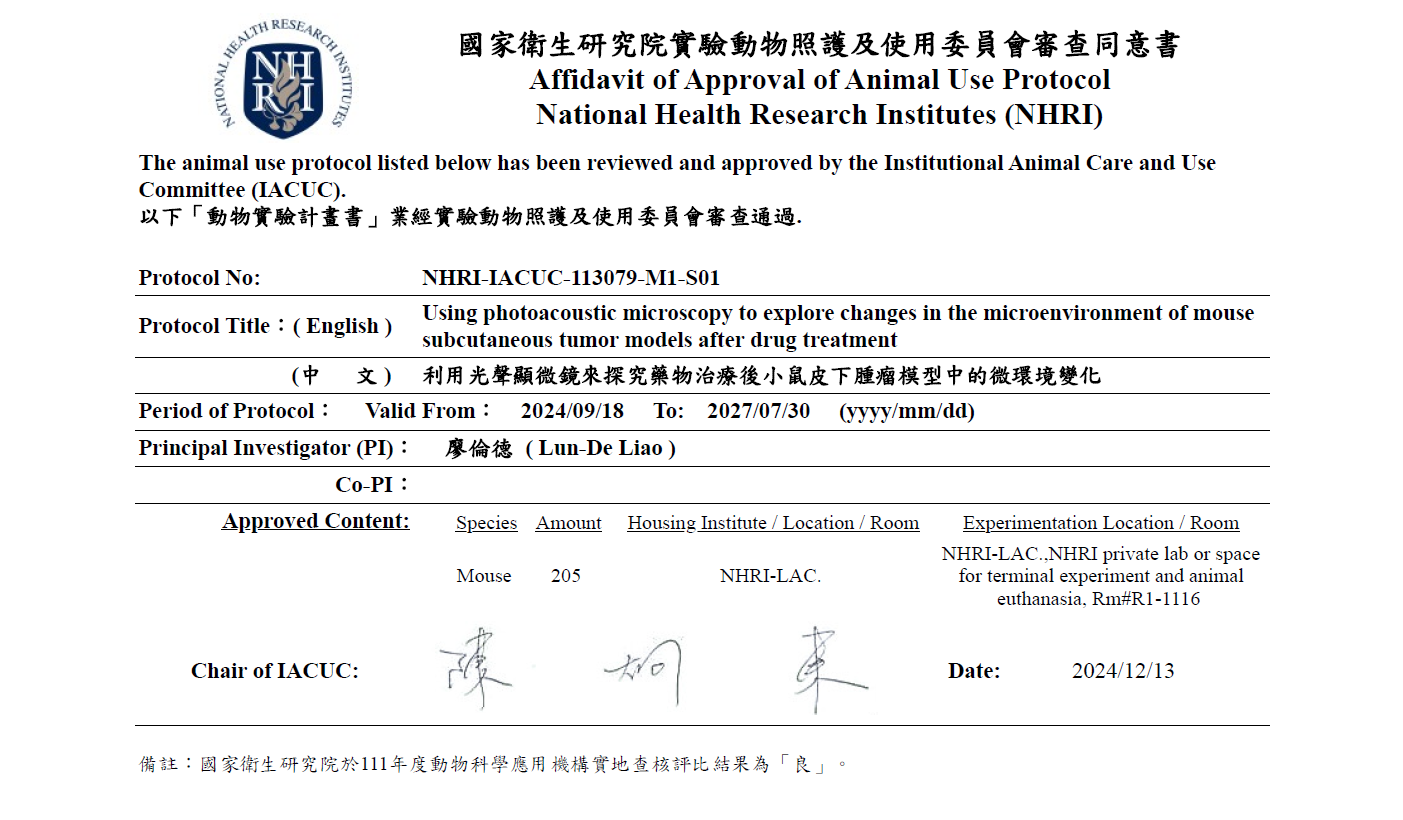


**References**

(1) Chien, W.-C.; Cheng, P.-H.; Cheng, X.-J.; Chuang, C.-C.; Huang, Y.-T.; Ts, A.; Liu, C.-H.; Lu, Y.-J.; Wu, K. C.-W. MCP-1-functionalized, core–shell gold nanorod@ iron-based metal–organic framework (MCP-1/GNR@ MIL-100 (Fe)) for photothermal therapy. *ACS Appl. Mater. Interfaces* **2021**, *13* (44), 52092-52105.

(2) Marshall, R. J.; Griffin, S. L.; Wilson, C.; Forgan, R. S. Stereoselective Halogenation of Integral Unsaturated C-C Bonds in Chemically and Mechanically Robust Zr and Hf MOFs. *Chem. Eur. J.* **2016**, *22* (14), 4870-4877. DOI: 10.1002/chem.201505185 From NLM.

(3) Marshall, R. J.; Richards, T.; Hobday, C. L.; Murphie, C. F.; Wilson, C.; Moggach, S. A.; Bennett, T. D.; Forgan, R. S. Postsynthetic bromination of UiO-66 analogues: altering linker flexibility and mechanical compliance. *Dalton Trans.* **2016**, *45* (10), 4132-4135.

(4) Marshall, R. J.; Griffin, S. L.; Wilson, C.; Forgan, R. S. Stereoselective Halogenation of Integral Unsaturated C‐C Bonds in Chemically and Mechanically Robust Zr and Hf MOFs. *Chemistry–A European Journal* **2016**, *22* (14), 4870-4877.

(5) Chuang, Y.-C.; Hsia, Y.; Chu, C.-H.; Maharajan, S.; Hsu, F.-C.; Lee, H.-L.; Chiou, J. F.; Ch’ang, H.-J.; Liao, L.-D.; Lo, L.-W. Photothermal temperature-modulated cancer metastasis harnessed using proteinase-triggered assembly of near-infrared II photoacoustic/photothermal nanotheranostics. *ACS Appl. Mater. Interfaces* **2024**, *16* (31), 40611-40627.

(6) Wang, Y.; Jhang, D.-F.; Tsai, C.-H.; Chiang, N.-J.; Tsao, C.-H.; Chuang, C.-C.; Chen, L.-T.; Chang, W.-S. W.; Liao, L.-D. In vivo assessment of hypoxia levels in pancreatic tumors using a dual-modality ultrasound/photoacoustic imaging system. *Micromachines* **2021**, *12* (6), 668.

(7) Leng, H.; Wang, Y.; Jhang, D.-F.; Chu, T.-S.; Tsao, C.-H.; Tsai, C.-H.; Giamundo, S.; Chen, Y.-Y.; Liao, K.-W.; Chuang, C.-C. Characterization of a fiber bundle-based real-time ultrasound/photoacoustic imaging system and its in vivo functional imaging applications. *Micromachines* **2019**, *10* (12), 820.

(8) Liu, D.; Lu, K.; Poon, C.; Lin, W. Metal–organic frameworks as sensory materials and imaging agents. *Inorg. Chem.* **2014**, *53* (4), 1916-1924.

(9) Bulte, J. W.; Kraitchman, D. L. Iron oxide MR contrast agents for molecular and cellular imaging. *NMR in Biomedicine: An International Journal Devoted to the Development and Application of Magnetic Resonance In Vivo* **2004**, *17* (7), 484-499.

(10) Kim, D.; Park, S.; Lee, J. H.; Jeong, Y. Y.; Jon, S. Antibiofouling polymer-coated gold nanoparticles as a contrast agent for in vivo X-ray computed tomography imaging. *Journal of the American Chemical Society* **2007**, *129* (24), 7661-7665.

(11) Furukawa, H.; Cordova, K. E.; O’Keeffe, M.; Yaghi, O. M. The chemistry and applications of metal-organic frameworks. *Science* **2013**, *341* (6149), 1230444.

(12) Alkilany, A. M.; Murphy, C. J. Toxicity and cellular uptake of gold nanoparticles: what we have learned so far? *J. Nanopart. Res.* **2010**, *12*, 2313-2333.

(13) Chowdhury, M. A. M etal‐organic‐frameworks for biomedical applications in drug delivery, and as MRI contrast agents. *J. Biomed. Mater. Res. Part A* **2017**, *105* (4), 1184-1194.

(14) Wang, G. D.; Chen, H.; Tang, W.; Lee, D.; Xie, J. Gd and Eu co-doped nanoscale metal–organic framework as a T1–T2 dual-modal contrast agent for magnetic resonance imaging. *Tomography* **2016**, *2* (3), 179.

(15) DeKrafft, K. E.; Boyle, W. S.; Burk, L. M.; Zhou, O. Z.; Lin, W. Zr-and Hf-based nanoscale metal–organic frameworks as contrast agents for computed tomography. *J. Mater. Chem.* **2012**, *22* (35), 18139-18144.

(16) Rieter, W. J.; Taylor, K. M.; An, H.; Lin, W.; Lin, W. Nanoscale metal− organic frameworks as potential multimodal contrast enhancing agents. *Journal of the American Chemical Society* **2006**, *128* (28), 9024-9025.

(17) Tian, C.; Zhu, L.; Lin, F.; Boyes, S. G. Poly (acrylic acid) bridged gadolinium metal–organic framework–gold nanoparticle composites as contrast agents for computed tomography and magnetic resonance bimodal imaging. *ACS Appl. Mater. Interfaces* **2015**, *7* (32), 17765-17775.

(18) Gielen, J. L.; De Schepper, A. M.; Vanhoenacker, F.; Parizel, P. M.; Wang, X. L.; Sciot, R.; Weyler, J. Accuracy of MRI in characterization of soft tissue tumors and tumor-like lesions. A prospective study in 548 patients. *Eur. Radio.* **2004**, *14*, 2320-2330.

(19) Gargiulo, S.; Albanese, S.; Mancini, M. State‐of‐the‐Art Preclinical Photoacoustic Imaging in Oncology: Recent Advances in Cancer Theranostics. *Contrast Media Mol. Imaging* **2019**, *2019* (1), 5080267.

(20) Cai, W.; Chen, X. Multimodality molecular imaging of tumor angiogenesis. *J. Nucl. Med.* **2008**, *49* (Suppl 2), 113S-128S.

(21) Li, M.-L.; Oh, J.-T.; Xie, X.; Ku, G.; Wang, W.; Li, C.; Lungu, G.; Stoica, G.; Wang, L. V. Simultaneous molecular and hypoxia imaging of brain tumors in vivo using spectroscopic photoacoustic tomography. *Proc. IEEE* **2008**, *96* (3), 481-489.

(22) Gong, T.; Li, Y.; Lv, B.; Wang, H.; Liu, Y.; Yang, W.; Wu, Y.; Jiang, X.; Gao, H.; Zheng, X. Full-process radiosensitization based on nanoscale metal–organic frameworks. *ACS Nano* **2020**, *14* (3), 3032-3040.

(23) Lusic, H.; Grinstaff, M. W. X-ray-Computed Tomography Contrast Agents. *Chem. Rev.* **2013**, *113* (3), 1641-1666. DOI: 10.1021/cr200358s.

(24) Liu, Y.; Li, J.; Chen, M.; Chen, X.; Zheng, N. Palladium-based nanomaterials for cancer imaging and therapy. *Theranostics* **2020**, *10* (22), 10057-10074. DOI: 10.7150/thno.45990 From NLM.

(25) Cho, E. C.; Glaus, C.; Chen, J.; Welch, M. J.; Xia, Y. Inorganic nanoparticle-based contrast agents for molecular imaging. *Trends Mol. Med.* **2010**, *16* (12), 561-573. DOI: 10.1016/j.molmed.2010.09.004 (acccessed 2025/04/09).

(26) Nadolski, G. J.; Stavropoulos, S. W. Contrast alternatives for iodinated contrast allergy and renal dysfunction: Options and limitations. *Journal of Vascular Surgery* **2013**, *57* (2), 593-598. DOI: https://doi.org/10.1016/j.jvs.2012.10.009.

(27) Hasebroock, K. M.; and Serkova, N. J. Toxicity of MRI and CT contrast agents. *Expert Opin. Drug Metab. Toxicol.* **2009**, *5* (4), 403-416. DOI: 10.1517/17425250902873796.

(28) Kumar, P.; Anand, B.; Tsang, Y. F.; Kim, K.-H.; Khullar, S.; Wang, B. Regeneration, degradation, and toxicity effect of MOFs: Opportunities and challenges. *Environ. Res.* **2019**, *176*, 108488.

(29) Kumar, S.; Jain, S.; Nehra, M.; Dilbaghi, N.; Marrazza, G.; Kim, K.-H. Green synthesis of metal–organic frameworks: A state-of-the-art review of potential environmental and medical applications. *Coord. Chem. Rev.* **2020**, *420*, 213407.

(30) Cai, W.; Chen, K.; Li, Z.-B.; Gambhir, S. S.; Chen, X. Dual-function probe for PET and near-infrared fluorescence imaging of tumor vasculature. *Journal of Nuclear Medicine* **2007**, *48* (11), 1862-1870.

(31) Zhang, C.; Sun, W.; Wang, Y.; Xu, F.; Qu, J.; Xia, J.; Shen, M.; Shi, X. Gd-/CuS-loaded functional nanogels for MR/PA imaging-guided tumor-targeted photothermal therapy. *ACS Appl. Mater. Interfaces* **2020**, *12* (8), 9107-9117.

(32) Song, J.; Zheng, J.; Li, P.; Lu, X.; Zhu, G.; Shen, P. An effective multimodal image fusion method using MRI and PET for Alzheimer's disease diagnosis. *Front. Digital Health* **2021**, *3*, 637386.

(33) Orza, A.; Yang, Y.; Feng, T.; Wang, X.; Wu, H.; Li, Y.; Yang, L.; Tang, X.; Mao, H. A nanocomposite of Au‐AgI core/shell dimer as a dual‐modality contrast agent for x‐ray computed tomography and photoacoustic imaging. *Med. Phys.* **2016**, *43* (1), 589-599.
